# Benchmarking of deep learning algorithms for 3D instance segmentation of confocal image datasets

**DOI:** 10.1371/journal.pcbi.1009879

**Published:** 2022-04-14

**Authors:** Anuradha Kar, Manuel Petit, Yassin Refahi, Guillaume Cerutti, Christophe Godin, Jan Traas

**Affiliations:** 1 Laboratoire RDP, Université de Lyon 1, ENS-Lyon INRAE, INRIA, CNRS, UCBL, Lyon, France; 2 Institut du Cerveau–Paris Brain Institute, Paris, France; 3 Université de Reims Champagne Ardenne, INRAE, FARE, UMR A 614, Reims, France; European Molecular Biology Laboratory, UNITED KINGDOM

## Abstract

Segmenting three-dimensional (3D) microscopy images is essential for understanding phenomena like morphogenesis, cell division, cellular growth, and genetic expression patterns. Recently, deep learning (DL) pipelines have been developed, which claim to provide high accuracy segmentation of cellular images and are increasingly considered as the state of the art for image segmentation problems. However, it remains difficult to define their relative performances as the concurrent diversity and lack of uniform evaluation strategies makes it difficult to know how their results compare. In this paper, we first made an inventory of the available DL methods for 3D cell segmentation. We next implemented and quantitatively compared a number of representative DL pipelines, alongside a highly efficient non-DL method named MARS. The DL methods were trained on a common dataset of 3D cellular confocal microscopy images. Their segmentation accuracies were also tested in the presence of different image artifacts. A specific method for segmentation quality evaluation was adopted, which isolates segmentation errors due to under- or oversegmentation. This is complemented with a 3D visualization strategy for interactive exploration of segmentation quality. Our analysis shows that the DL pipelines have different levels of accuracy. Two of them, which are end-to-end 3D and were originally designed for cell boundary detection, show high performance and offer clear advantages in terms of adaptability to new data.

## Author summary

In recent years, a number of deep learning (DL) algorithms based on computational neural networks have been developed, which claim to achieve high accuracy and automatic segmentation of three-dimensional (3D) microscopy images. Although these algorithms have received considerable attention in the literature, it is difficult to evaluate their relative performances, while it remains unclear whether they really perform better than other, more classical segmentation methods. To clarify these issues, we performed a detailed, quantitative analysis of a number of representative DL pipelines for cell instance segmentation from 3D confocal microscopy image datasets. We developed a protocol for benchmarking the performances of such DL-based segmentation pipelines using common training and test datasets, evaluation metrics, and visualizations. Using this protocol, we evaluated and compared 4 different DL pipelines to identify their strengths and limitations. A high performance non-DL method was also included in the evaluation. We show that DL pipelines may show significant differences in their performances depending on their model architecture and pipeline components but overall show excellent adaptability to unseen data. We also show that our benchmarking protocol could be extended to a variety of segmentation pipelines and datasets.

## Introduction

The use of three-dimensional (3D) quantitative microscopy has become essential for understanding morphogenesis at cellular resolution, including cell division and growth as well as the regulation of gene expression [1]. In this context, image segmentation to identify individual cells in large datasets is a critical step. Segmentation methods broadly belong to 2 types, namely “semantic segmentation” in which each pixel within an image is associated with one of the predefined categories of objects present in the image. The other type, which is of interest in this paper, is “instance segmentation” [2]. This type of method goes one step further by associating each pixel with an independent object within the image. Segmenting cells from microscopy images falls within this second type of problem. It involves locating the cell contours and cell interiors such that each cell within the image may be identified as an independent entity [3]. High accuracy cell instance segmentation is essential to capture significant biological and morphological information such as cell volumes, shapes, growth rates, and lineages [4].

A number of computational approaches have been developed for instance segmentation (e.g., [1,5–7] such as, for example, the commonly used watershed, graph partitioning, and gradient-based methods. In watershed approaches, seed regions are first detected using criteria like local intensity minima or user provided markers. Starting from the seed locations, these techniques group neighboring pixels by imposing similarity measures until all the individual regions are identified. In graph partitioning, the image is treated as a graph, with the image pixels as its vertices. Subsequently pixels with similar characteristics are clustered into regions also called superpixels. Superpixels represent a group of pixels sharing some common characteristics such as pixel intensity. In some graph-based approaches such as [8–10], superpixels are first estimated by oversegmenting an image followed by graph partitioning to aggregate these superpixels into efficiently segmented regions of the image. Gradient-based methods use edge or region descriptors to drive a predefined contour shape (usually rectangles or ellipses) and progressively fit them to accurate object boundaries, based on local intensity gradients [11,12].

Common challenges faced by these segmentation methods arise in low-contrast images containing fuzzy cell boundaries. This might be due to the presence of nearby tissue structures as well as anisotropy of the microscope that perturb signal quality, poor intensity in deeper cell layers as well as blur and random intensity gradients arising from varied acquisition protocols [13,14]. Some errors can also be due to the fact that cell wall membrane markers are not homogenous at tissue and organ level: In some regions, the cell membrane is very well marked, resulting in an intense signal, while in the other regions, this may not be the case. These different problems lead to segmentation errors such as incorrect cell boundary estimation, single cell regions mistakenly split into multiple regions (oversegmentation), or multiple cell instances fusing to produce a condensed region (undersegmentation).

In recent years, a number of computational approaches based on large neural networks (commonly known as deep learning or DL) [15] have been developed for image segmentation [16–18]. The key advantages of DL-based segmentation algorithms include automatic identification of image features, high segmentation accuracy, requirement of minimum human intervention (after the training phase), no need for manual parameter tuning during prediction, and very fast inferential capabilities. These DL algorithms are made of computational units (“neurons”), which are organized into multiple interconnected layers. For training a network, one needs to provide input training data (e.g., images) and the corresponding target output (ground truth). Each network layer transforms the input data from the previous level into a more abstract feature map representation for the next level. The final output of the network is compared with the ground truth using a loss (or cost) function. Learning in a neural network involves repeating this process and automated tuning of the network parameters multiple times. By passing the full set of training data through the DL network a number of times (also termed “epochs”), the network estimates the optimal mapping function between the input and the target or ground truth data. The number of epochs can be in the order of hundreds to thousands depending on the type of data and the network. The training will run until the training error is minimized. Thereafter, in a “recognition” phase, the neural network with these learned parameters can be used to identify patterns in previously unseen data.

In case of application of DL for image segmentation, the training and inference process is identical as above. The input data comprise raw images (grayscale or RGB), and the ground truth data are composed of highly precise segmentations of these input images where the desired regions are labeled.

**Fig 1:**
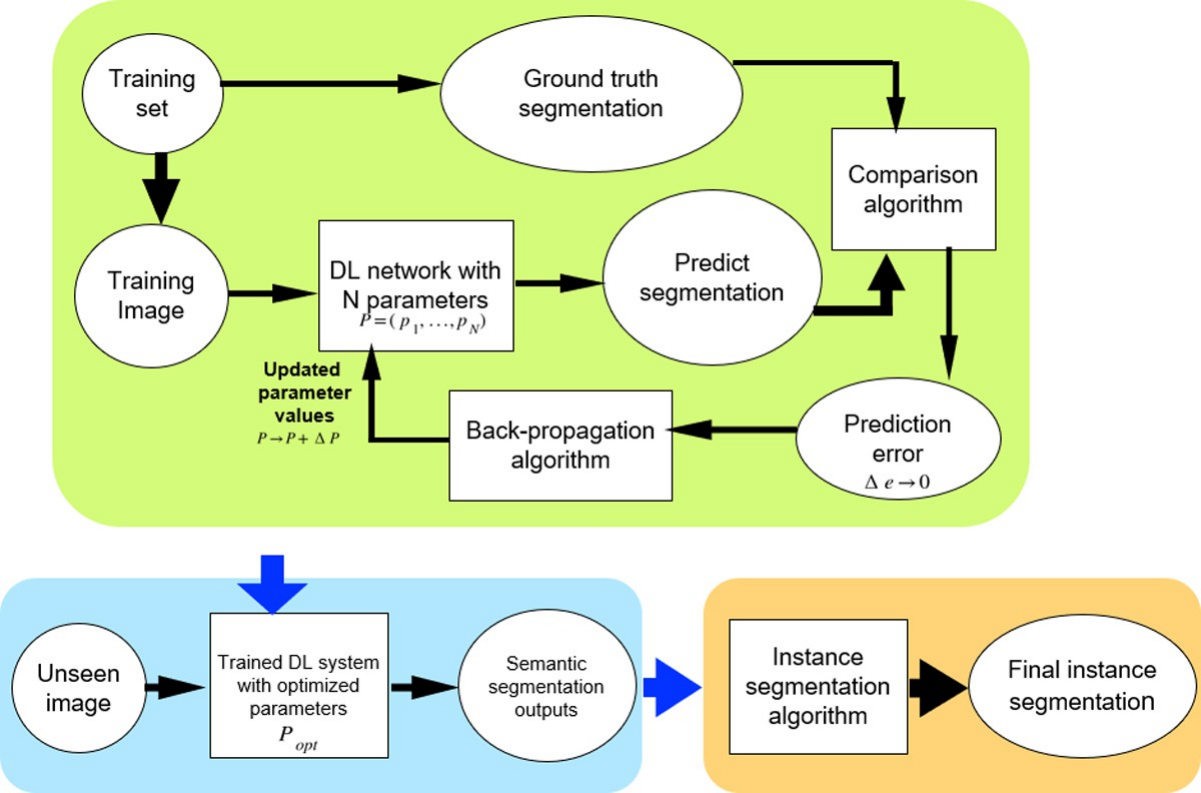
Generic workflow of a DL-based image segmentation pipeline. The DL network is first trained to produce a semantic segmentation which corresponds as closely as possible to a given ground truth. The trained network is then used to segment unseen images. The resulting semantic segmentation is then further processed to obtain the final instance segmentation. DL, deep learning.

Instance segmentation using DL is a challenging task especially for 3D data due to large computational time and memory requirements for extracting individual object instances from 3D data [19,20]. Therefore, the current trend in DL-based segmentation methods is to proceed in 2 steps. First, deep networks are used to provide high-quality semantic segmentation outputs. This involves the extraction of several classes of objects within an image such as cell boundaries, cell interiors, and background. These DL outputs are then used with traditional segmentation methods to achieve the final high accuracy and automatic instance segmentation even in images with noise and poor signal quality [21]. A generic workflow of such a DL-based instance segmentation process is shown in Fig 1.

In contemporary DL literature, 2 types of architecture for segmentation are commonly used: the ones based on the UNet/residual UNet network [22,23] and the approaches using the region proposal networks (RPNs) or Region-based Convolutional Neural Networks (RCNNs) [24]. We will only briefly present both the general properties of both types of networks. The UNet [22] has a symmetric DL architecture. One part (called encoder) extracts the image features, and the other part (named decoder) combines the features and spatial information to obtain the semantic segmentation, for example, cell boundaries, cell body, and image background. In order to obtain the instance segmentation, this is followed by methods such as watershed or graph partitioning. Examples of UNet-based two-dimensional (2D) and 3D segmentation algorithms include, e.g., [21,25,26].

Besides UNet, the other state-of-the-art DL architecture is a RCNN such as Mask R-CNN or MRCNN [27]. MRCNN differs from UNet as the former includes modules for object detection and classification unlike the UNets. MRCNN has been used for high accuracy and automatic segmentation of microscopy images in several works (e.g., [28,29]).

There currently exists a large number of DL pipelines (we have identified and reviewed up to 35 works in the last 5 years in S4 File), where variants of both the above architectures are used to address specific challenges in segmentation such as sparse datasets, availability of partial ground truths, temporal information, etc. (see S4 File for a more extensive review). However, the diversity of the currently available pipelines and inconsistent use of segmentation accuracy metrics makes it difficult to characterize and evaluate their relative performance based on the literature. The presence of such diversity has motivated several benchmarking studies such as [30,31]. Ulman and colleagues [30] describe a thorough evaluation of 21 cell tracking pipelines. One of these includes a 3D UNet DL system for segmentation. This study evaluates the capability of the methods to segment and track correctly different types of data (optical and fluorescent imaging and single and densely packed cells). Leal-Taixé and colleagues [31] compare 4 epithelial cell tracking algorithms using 8 time-lapse series of epithelial cell images. Although both papers highlight the importance of accurate image segmentation and underline the performance of DL, the characterization of the method errors is rather focused on the cell tracking part. In this paper, we focus in detail on the segmentation itself, comparing extensively and quantitatively the capacity of a number of selected DL protocols to accurately segment 3D images. To do so, we retrained the DL systems on a common benchmark 3D dataset and analyzed segmentation characteristics of each pipeline at cellular resolution. The pipelines used here are based on either UNet or RCNN architectures and were selected from the literature based on the following criteria. (i) First, as the focus of this work is on 3D confocal datasets, the pipelines are built for 3D instance segmentation of static images. Analyses of temporal information or specific architectures for cell or particle tracking are not included as these are extensively covered in [30,31]. (ii) Next, the pipeline implementations including pre- and postprocessing methods are available in open-source repositories. (iii) To ensure that the pipelines are reproducible properly on other machines, the training dataset used originally by the authors are available publicly. (iv) Last, the DL pipelines are trainable with new datasets. Based on these criteria, we identified 4 pipelines ([21,24,26,32]), which we further describe below.

The first pipeline is an adapted version of Plantseg [26], which can be trained using 3D images composed of voxels. It uses a variant (see Materials and methods section) of 3D UNet called residual 3D-UNet [23] for the prediction of cell boundaries in 3D, resulting in a semantic segmentation. These are then used in a postprocessing step for estimating the final instance segmentation using graph partitioning. Examples of graph partitioning include GASP [33] and Multicut [10].

The second DL pipeline [21] comprises a 3D UNet, which can be trained using 3D confocal images (i.e., composed of voxels) for prediction of cell boundary, cell interior, and image background regions (as 3D images). These semantic outputs of the 3D UNet are then used to generate a seed image for watershed-based postprocessing. Seeds in watershed segmentation indicate locations within images from where growing of connected regions starts in the image watershed map. The seed images produced from the UNet outputs in this pipeline are therefore used to perform 3D watershed and obtain the final segmentation output.

The third pipeline is adapted from Cellpose [32]. It uses a residual 2D-UNet architecture, which should be trained using 2D images (composed of pixels). The 2D trained UNet predicts horizontal (X) and vertical (Y) vector gradients of pixel values, or flows, along with a pixel probability map (indicating whether pixels are inside or outside of the cell regions) for each 2D image. By following the vector fields, the pixels corresponding to each cell region are clustered around the cell center. This is how 2D gradients in XY, YZ, and ZX planes are estimated. These 6 gradients are averaged together to find 3D vector gradients. These 3D gradients are used to estimate the cell regions in 3D.

**Fig 2:**
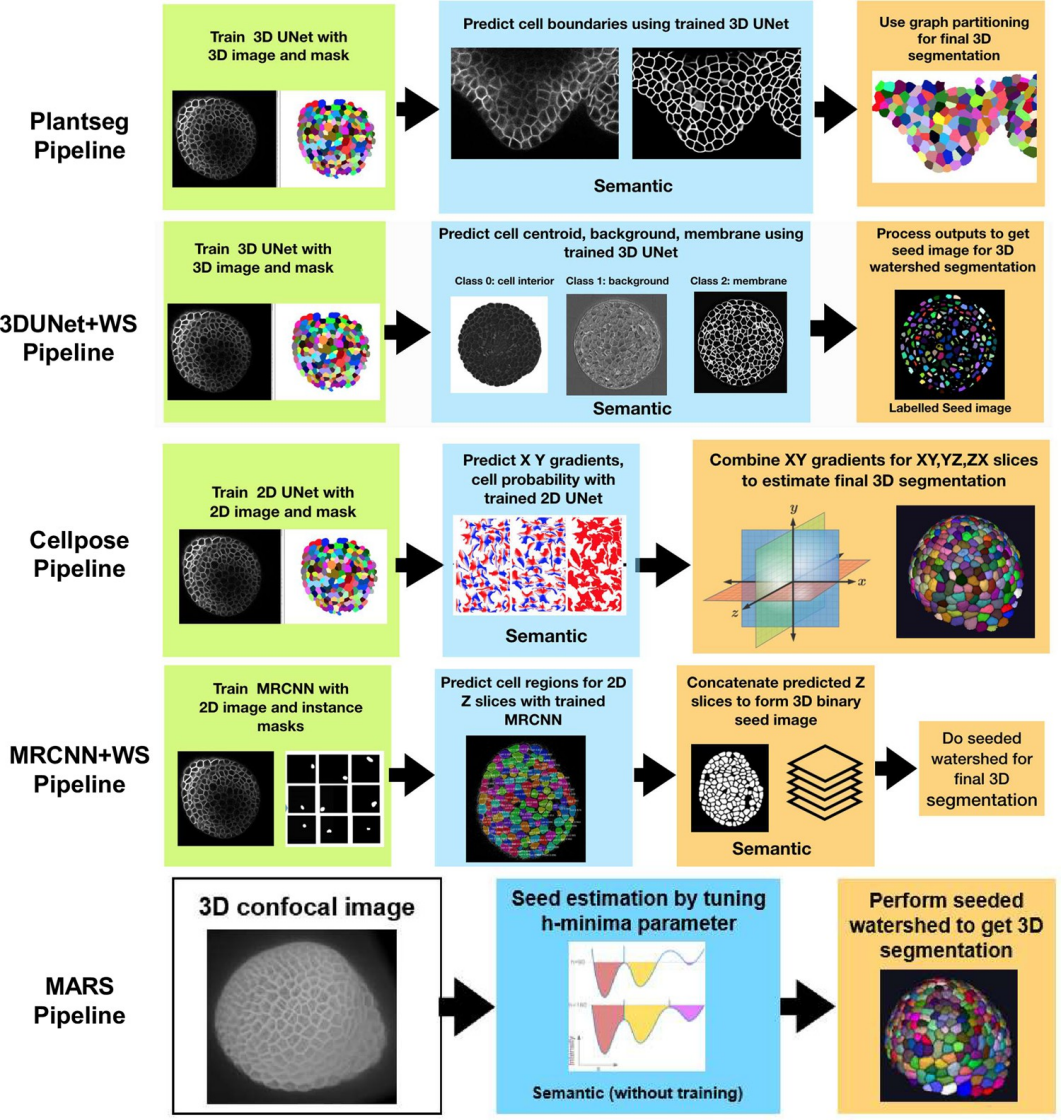
Displaying all the 3D segmentation pipelines together. The green colored boxes indicate the training process for the respective pipeline. The blue boxes indicate the predicted, semantic segmentations generated by the trained DL algorithms, and the orange boxes indicate phases of postprocessing, leading to the final instance segmentation. The MARS pipeline doesn’t include a training or postprocessing step, but parameter tuning is required. 3D, three-dimensional.

The fourth DL pipeline is adapted by the authors of this paper from the well-documented open Mask R-CNN repository [24], and the 3D segmentation concept using this model is inspired from [34]. For the Mask R-CNN–based segmentation, a hybrid approach is adopted as shown in Fig 2. The pipeline uses a MRCNN algorithm, which is trained using 2D image data to predict which pixels belong to cell areas and which do not in each Z slice of a 3D volume leading to a semantic segmentation. Then, the Z slices containing the identified cell regions are stacked into a binary 3D seed image. The cell regions in this binary image are labeled using the connected component approach, where all voxels belonging to a cell are assigned a unique label. These labeled cell regions are used as seeds for watershed-based processing to obtain the final 3D instance segmentation.

A further aspect of investigation in this work is to observe how these DL pipelines compare to a classical non-DL pipeline in terms of segmentation accuracy. They were therefore compared with a watershed-based segmentation pipeline named MARS [35], which uses automatic seed detection and watershed segmentation. In the MARS pipeline, the minima of local intensity of the image detected by a h-minima operator is used to initiate seeds in the image which are then used for 3D watershed segmentation of cells. This pipeline therefore does not involve any model training component.

As these 5 segmentation pipelines have been developed and tested on different datasets and have been characterized using different evaluation metrics, it is difficult to directly compare their performance. For example, in the original papers, the Plantseg and UNet+WS pipelines were trained and designed for images having membrane stainings and therefore use a UNet-based boundary detection method. The Cellpose model was originally trained with diverse types of bio-images such as those with cytoplasmic and nuclear stains and microscopy images with and without fluorescent membrane markers. The Mask R-CNN adopted in this work was originally trained using images from cell nuclei.

Therefore, the first step of the benchmarking protocol was to train the 4 DL pipelines on a common 3D image dataset. We next tested all the 5 segmentation (DL and non-DL) pipelines on a common 3D test image dataset. This was followed by estimating and comparing their performance based on a common set of metrics. Through this protocol, we aimed to develop an efficient strategy for quantitative and in-depth comparison of any 3D segmentation pipeline that currently exists or is under development. Our results show clear differences in performance between the different pipelines and highlight the adaptability of the DL methods to unseen datasets.

## Results

### A benchmarking protocol for 3D segmentation pipelines

**Fig 3:**
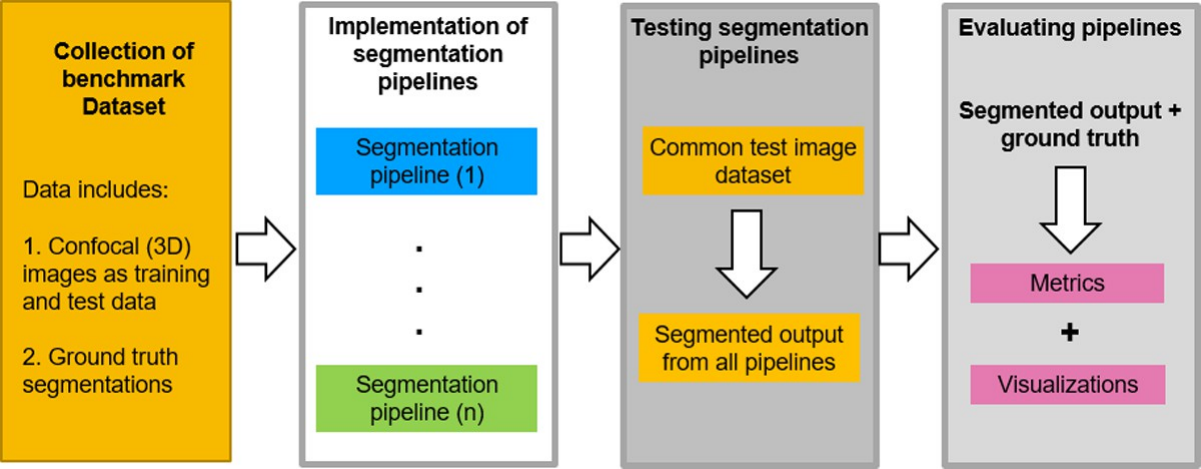
Schematic workflow of the benchmarking process. The evaluation of segmentation pipelines begins with the training of the DL models on a common training dataset (confocal images and ground truth). The training and postprocessing steps for each pipeline are reproduced in the exact way as defined in the respective papers or their repositories. Then, the 5 pipelines are tested on a common test set of images. The test dataset (Fig 4) contains both raw confocal images and their corresponding expert annotated ground truths, and, therefore, it is possible to assess the segmentation accuracy of the 5 pipelines by comparing segmentation output of each pipeline with the respective ground truth data. Finally, the relative accuracy of each method is evaluated using multiple strategies. DL, deep learning.

**Fig 4:**
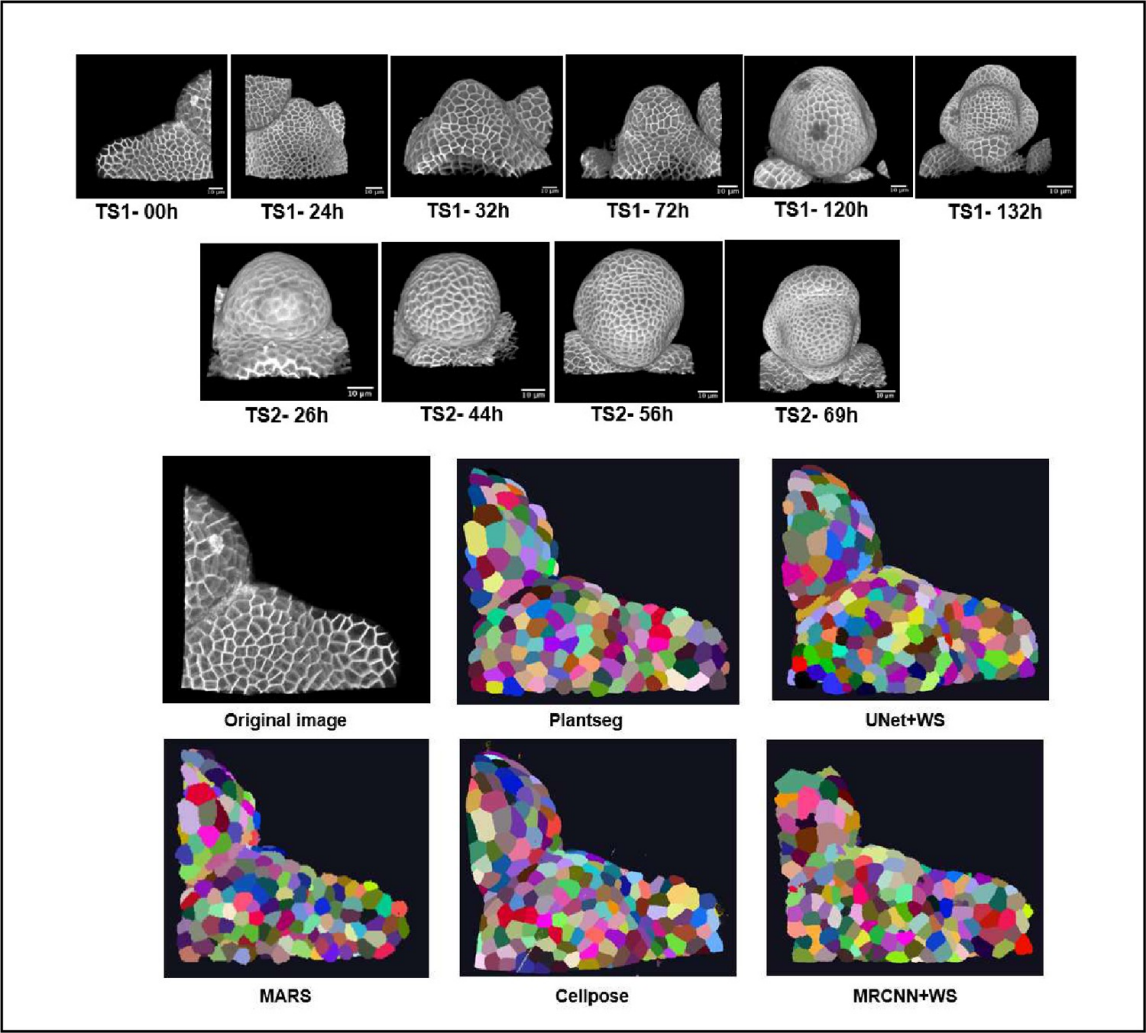
**(A)** The 2 test datasets containing a total of 10 confocal image stacks of 2 different *Arabidopsis* floral meristems. (B) A sample test stack (TS1-00H) and its segmentation by 5 segmentation pipelines.

The benchmarking workflow for the segmentation pipelines adapted in this paper is shown in the schematic diagram of Fig 3. All 4 DL pipelines were trained following the specifications of respective pipelines (more details in Materials and methods section) as given in their repositories. For training, we used a common set composed of 124 3D original stacks of confocal images from *Arabidopsis* shoot apical meristems (SAMs) and their ground truth segmentations, which are publicly available, as described in [36]. This is one of the more extensive 3D confocal sets with ground truth publicly available. The trained networks were used to segment 2 test datasets of floral meristem images (Fig 4A) described in [45] (see Materials and methods section) and for which ground truths were available as well. Sample results from these pipelines on one test stack (TS1-00h) are shown in Fig 4B.

For MARS, a manual tuning of 3 parameter values is generally required to obtain optimal segmentation (h-minima and Gaussian smoothing sigma for image and that for seeds; see Materials and methods section for details on MARS parameters to tune). This can involve many trials before optimal segmentation is obtained and needs expert supervision. For the sake of comparison with the DL methods, which were only trained once, we therefore only used one set of parameters. First, the optimal MARS parameters were found for one 3D image, and these were then kept constant for the remaining images in the test set.

The other parts of the benchmarking workflow, which is testing and evaluation of the 5 segmentation pipelines through common strategies, metrics, and visualizations, are detailed below.

### Comparing the segmentation pipelines

We next compared the quality of the segmentations produced by the 5 pipelines. For this purpose, we adopted 3 different strategies (see also Materials and methods for details). First, we analyzed the quality of segmentation on overall stacks, including all cell layers. Next, the confocal stacks were split into different cellular layers (L1, L2, and inner), and the segmentation quality of the 5 pipelines for each layer was studied. Finally, we evaluated the segmentation quality on images with commonly occurring (artificially generated) aberrations. In each strategy, several metrics were used for quantitative assessment.

#### Strategy 1: Evaluating segmentation quality for entire image stacks

To estimate the segmentation quality of the outputs from the 5 pipelines, we used their segmented results and the corresponding ground truths of the test datasets. A volume-averaged Jaccard index (VJI) metric was used to estimate overlap between the predicted segmentations and ground truths. The VJI used here measures the degree of overlap averaged over the cell volume. In the VJI metric, the averaging over cell volume is done to avoid biases arising from the cell sizes on the standard JI. Also, metrics that identify the rates of over- and undersegmentation were applied (details of this metric is in the Materials and methods section). The rate of oversegmentation is the % of cells in the ground truth associated with multiple regions in the predicted segmentation. Conversely, the rate of undersegmentation is the percentage of cases where several regions in the ground truth are associated with a single cell in the predicted segmentation. Another estimate shown on Table 1 is the percentage of missing cells, which refer to the percentage of cells from the ground truth that are not in the predicted segmentation.

**Table 1:** Mean values (average over the 2 test datasets) of segmentation evaluation metrics.

Algorithms	VJI	Rate of oversegmentation (%)	Rate of undersegmentation (%)	Percentage of missing cells (%)
**Plantseg**	0.819	4.457	6.178	2.051
**3D UNet+ WS**	0.743	4.066	13.290	1.705
**Cellpose**	0.694	13.456	10.653	4.727
**Mask R-CNN+WS**	0.560	14.632	22.995	9.621
**MARS**	0.815	2.576	12.492	2.759

In MARS, the optimal parameter values are only determined for one image (h-minima = 2 and Gaussian smoothing sigma for image = 0.4).

VJI, volume-averaged Jaccard index.

**Fig 5:**
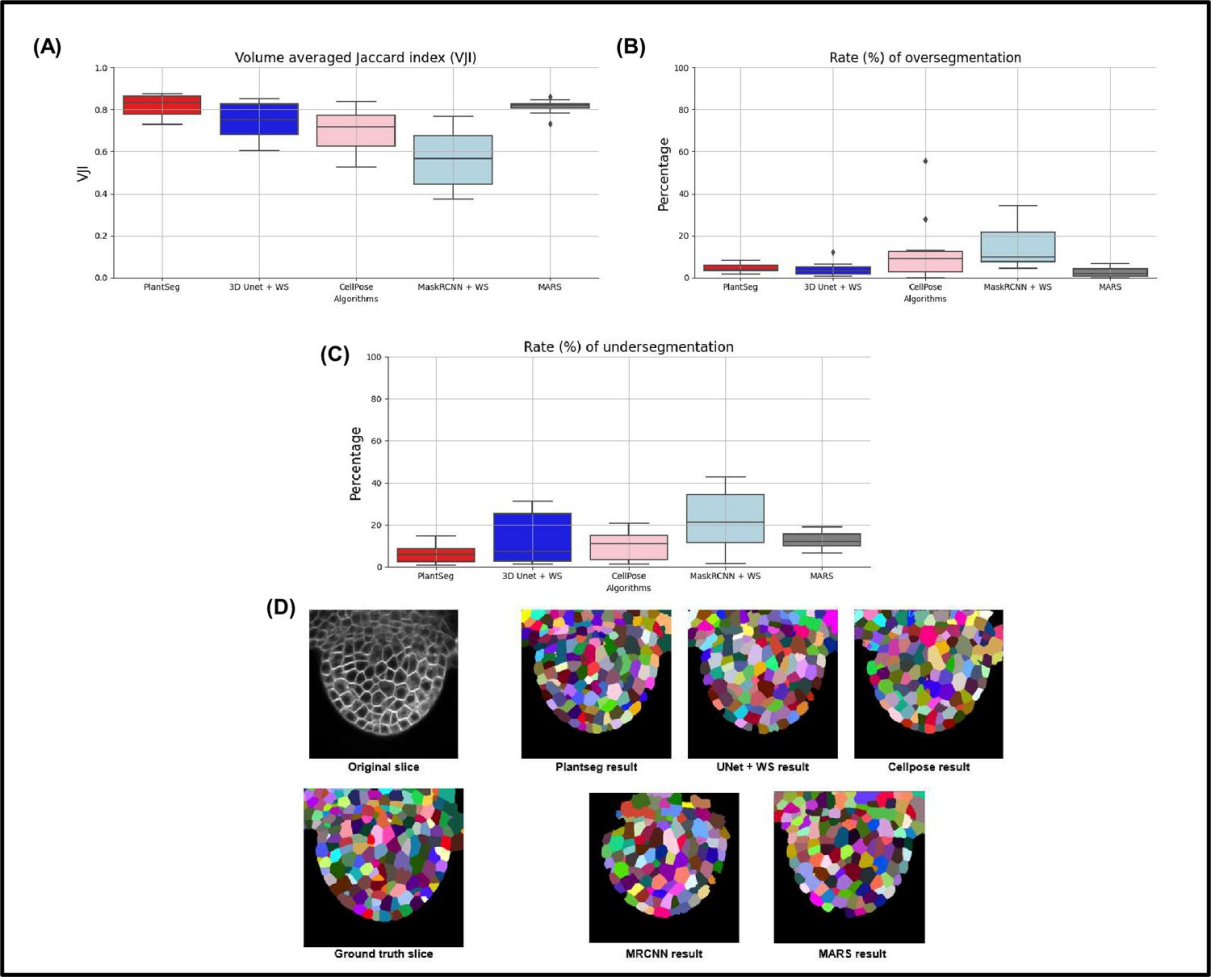
**(A)** Results of VJI metric from the 5 segmentation pipelines. Note that VJI is computed for each pair of segmented image/ ground truth image, and so the VJI statistics shown above are computed on the values of VJI of the 10 3D test images for each pipeline. **(B)** and **(C)** shows rates of over- and undersegmentation, which is computed using a segmented stack and corresponding ground truth stack as input. The distributions shown here are estimated over the results from the 2 test datasets TS1 and TS2. **(D)** Example segmentation results by 5 pipelines on a test image slice. 3D, three-dimensional; VJI, volume-averaged Jaccard index.

The results, summarized in Table 1 and Fig 5A–5D, reveal a number of differences between the pipelines. Sample results are shown in Fig 5D.

Among all the pipelines, Plantseg performs best as measured using the VJI metric values (Fig 5A), closely followed by MARS and then UNet+Watershed. Cellpose and, in particular, MRCNN+Watershed perform less well.

With respect to rates of oversegmentation (Fig 5B), The MARS and UNet+Watershed pipelines have lowest rates very closely followed by PlantSeg. The 2 hybrid pipelines Cellpose and MRCNN+Watershed have higher rates of oversegmentation. The rate of undersegmentation (Fig 5C) is higher for all the pipelines than the rate of oversegmentation, but Plantseg performs better than the others. Overall, we can conclude that the errors in the pipelines are mostly due to undersegmentation.

#### Strategy 2: Segmentation quality evaluation for different cell layers

Three-dimensional confocal stacks often show different levels of intensity and contrast in different cell depths. In particular, in the inner layers, the cell segmentation can be challenging. We therefore tested the performance of the pipelines for their capacity to segment the different cell layers.

**Fig 6:**
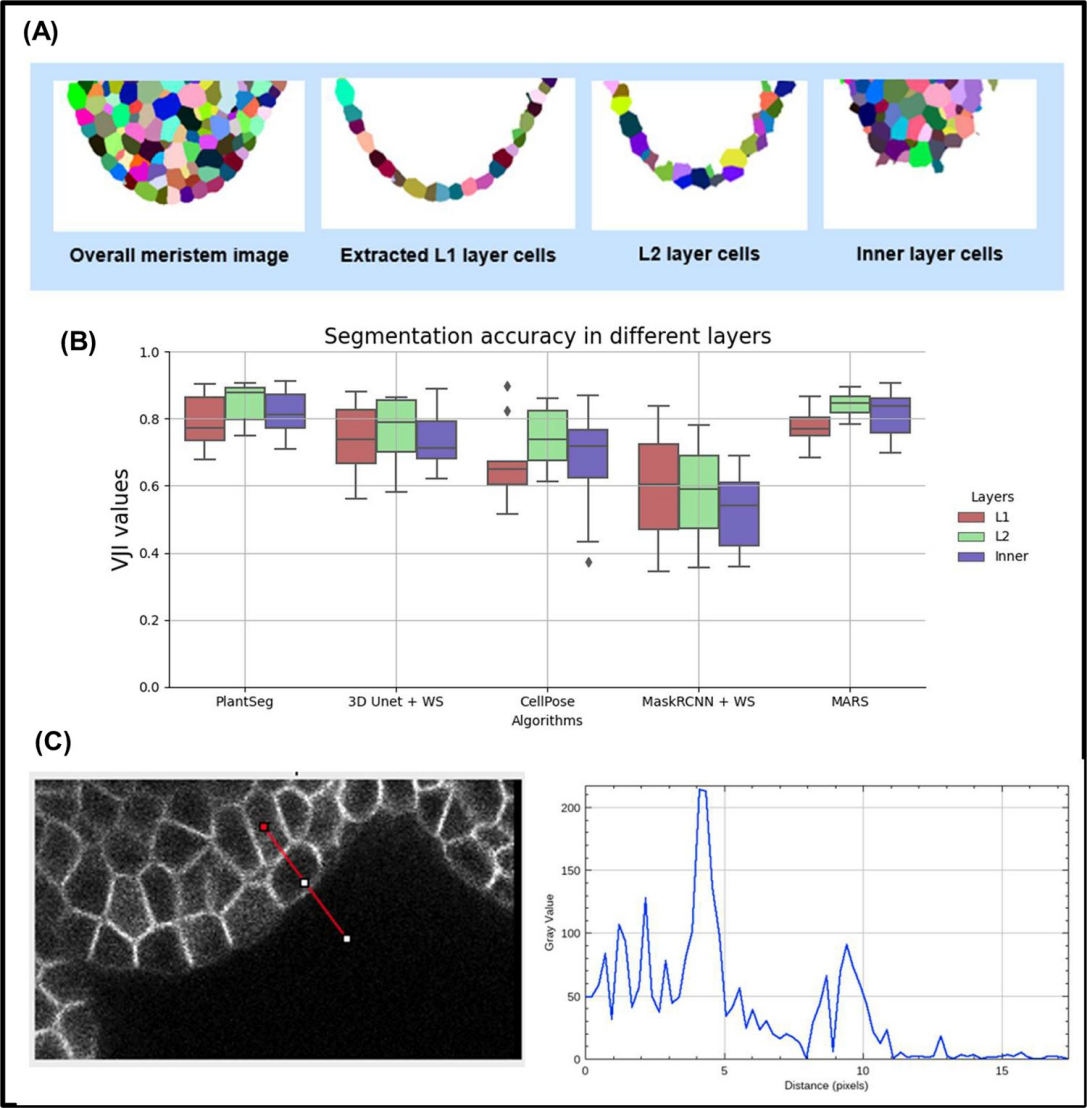
**(A)** Extracting L1, L2, and inner layers from an input segmented meristem image. **(B)** Estimating segmentation accuracy (VJI) for different cell layers. All stacks from the test dataset are used for this evaluation. **(C)** Boundary Intensities profile plot for outer and inner layer cells. The gray value at x = 0 on the plot on the left is the gray value of the image at the red point of the line segment drawn on the right image.

For identifying the layers from the ground truth segmented stacks, we carefully manually classify the cells in contact with the background into 3 classes: L1, L2, and inner layer. Then, an automatic procedure was applied to propagate the classes to the remaining cells. L2 cells were defined as cells in contact with L1 cells but not the background. Inner cells were defined as cells in contact with L2 cells exclusively (Fig 6A).

The variation of segmentation quality of the different pipelines was studied on the basis of the VJI values in the 3 cellular layers (Fig 6B).

Plantseg and MARS produce the most accurate segmentations in all the layers out of the 5 pipelines, and their accuracy doesn’t degrade significantly in the outer or inner layers. The VJI index of the UNet+Watershed pipeline is only slightly lower and nearly the same for all the layers. Cellpose and MRCNN+Watershed perform less well than the others, in all 3 layers, and MRCNN accuracy drops further in the innermost layer. However, with the exception of Mask R-CNN, the segmentation of the L2 layer is slightly better than that of the L1. This might be linked to the weak labeling of the outer membranes (Fig 6C), which is often observed.

It may be noted that this layer-wise analysis protocol is developed to study how the segmentation pipelines perform when going deeper into the tissue, i.e., as the image signal levels get weaker. This analysis is, therefore, also relevant for other tissue types as well, which involves imaging at different cell depths.

#### Strategy 3: Evaluating pipelines on synthetically modified images

Confocal images are often affected by effects such as noise, shadows, and motion blur, which tend to perturb the image signal. Especially in the inner layers, due to loss of optical signal and scattering, the images contain regions of very poor signal and distortions. In order to study the impact of these variations on the segmentation quality, the effects of noise, blur, and intensity variations were simulated on the test set of confocal images. The 5 segmentation algorithms were then applied to the modified images, and the VJI values and rates of under- or oversegmentations were estimated to observe and compare their robustness against these conditions.

**Fig 7:**
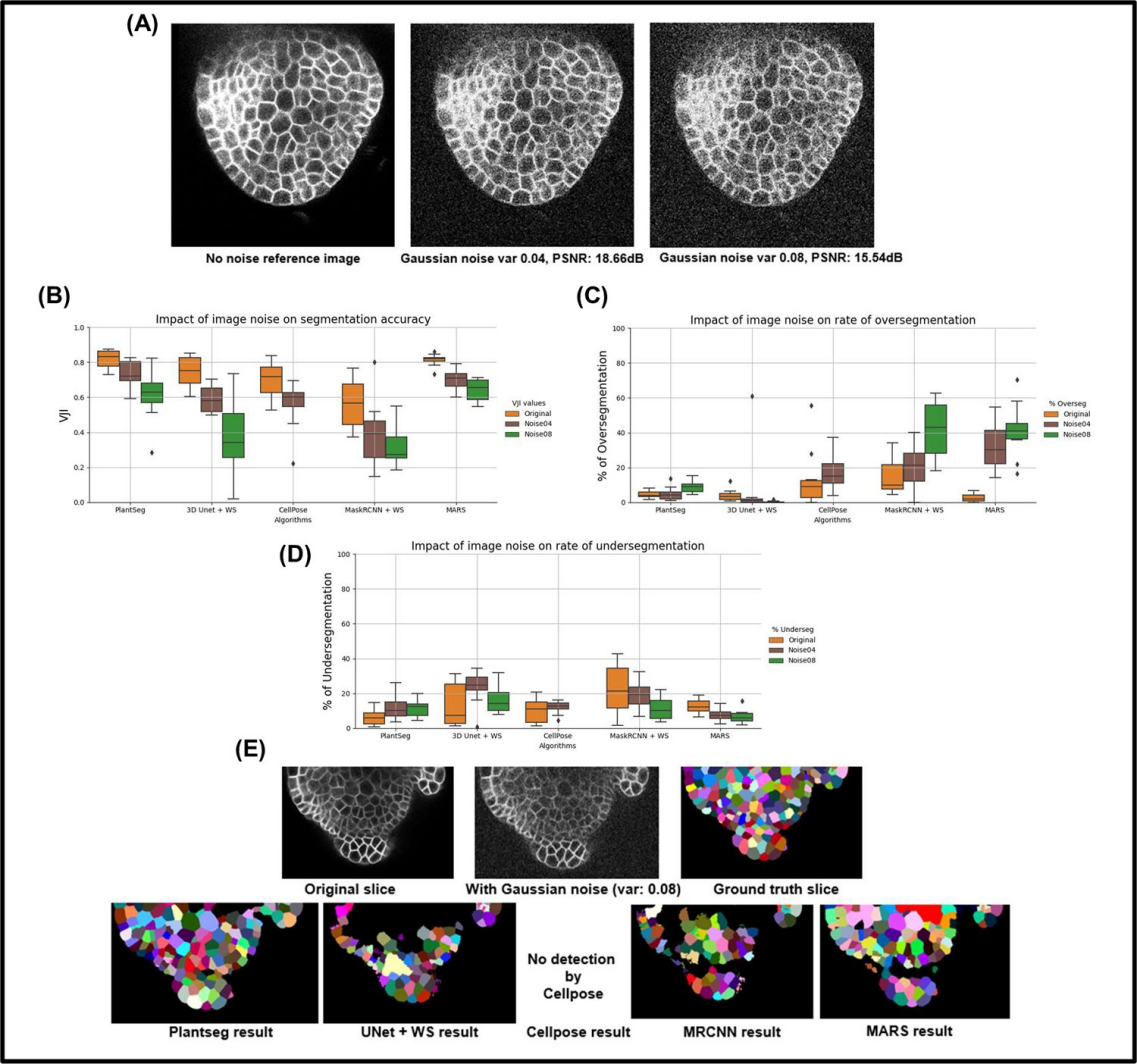
**(A)** A test image after applying Gaussian noise (var 0.04, 0.08). **(B)** Variation of segmentation accuracy (VJI) with 3 Gaussian noise variances. **(C)** Variation in rates of oversegmentation. **(D)** Variation in rates of undersegmentation. Note that for noise variance of 0.08, Cellpose is unable to identify cells. **(E)** Example results from the 5 pipelines under the impact of image noise (Gaussian noise variance 0.08). PSNR, peak signal-to-noise ratio; VJI, volume-averaged Jaccard index.

**Effect of image noise.** The electronic detector or amplifier of the imaging equipment can generate noise, which can be modeled using Gaussian statistics [37]. An image to which 2 different Gaussian noise levels corresponding to noise variances of 0.04 and 0.08 are added is shown in Fig 7A. At higher variance, the noise effect is increased as seen in the peak signal-to-noise ratio (PSNR) values.

The 5 pipelines behaved very differently under the impact of image noise as shown in Fig 7B. In particular, UNet+WS, CellPose, and MRCNN are very sensitive to Gaussian noise as their accuracy drops sharply when Gaussian noise variance is increased. At a noise variance of 0.08, Cellpose shows no detection. For MRCNN, higher noise leads to loss in identified cell regions, which results in large blob-like regions after watershed-based postprocessing, leading to higher undersegmentation (Fig 7D). The difference with Plantseg could be due to differences in the instance segmentation components. Plantseg uses graph partitioning, while UNet+WS and MRCNN pipelines use 3D watershed, although the seed identification criteria are different for them. MARS is most sensitive when it comes to oversegmentation (Fig 7C), while PlantSeg is more sensitive in terms of undersegmentation.

**Fig 8:**
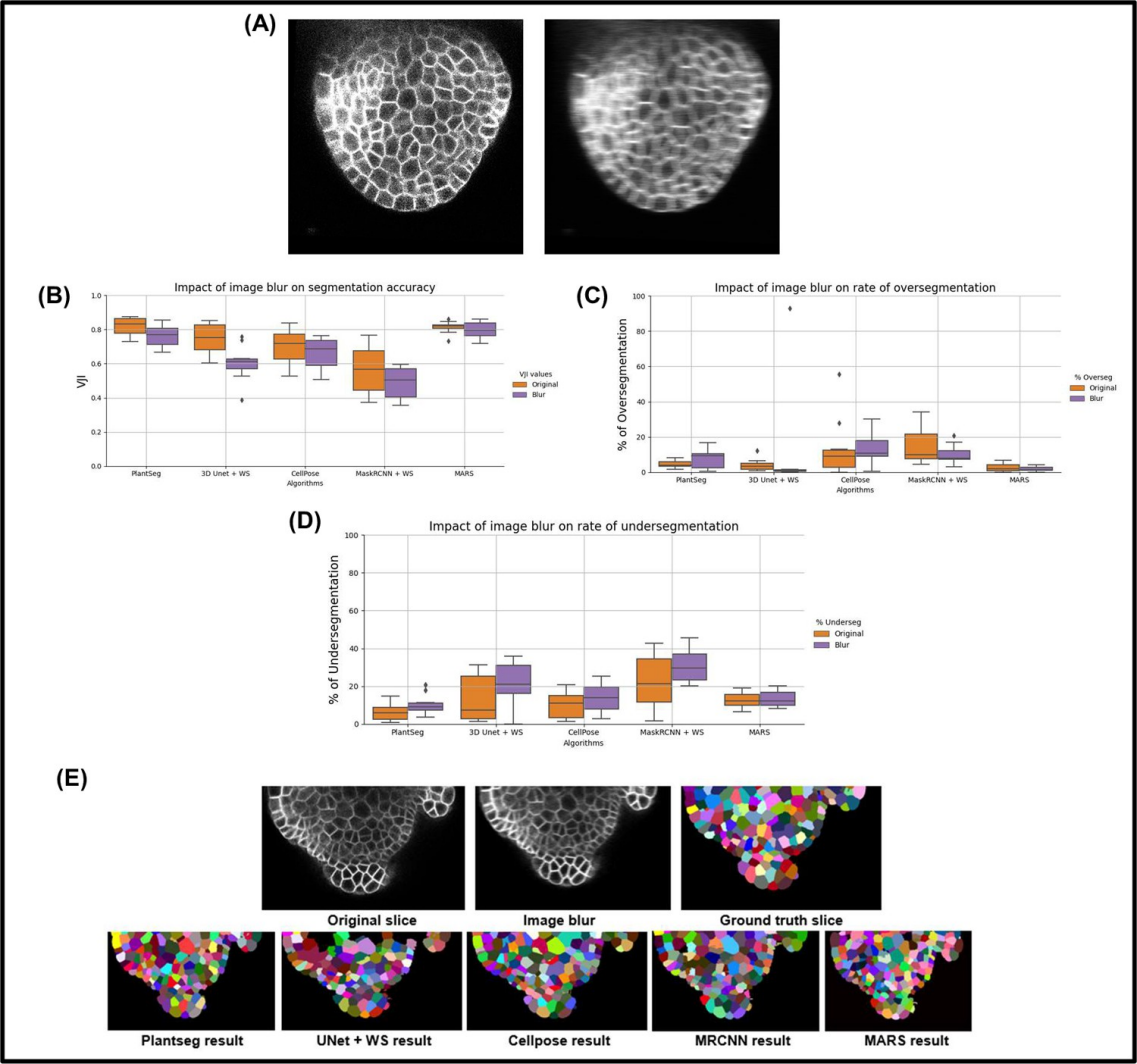
**(A)** Effect of blurring on an image. **(B)** Comparing segmentation accuracies of pipelines under the effect of image blur. **(C)** Comparing rates of oversegmentation. **(D)** Undersegmentations due to image blur. **(E)** Results from the 5 pipelines under the impact of image blur.

**Effect of image blur.** Blurring is common in images and can, for example, be caused by lens aberrations or optical diffraction in the imaging setup [38]. It can also be caused by motion of the objects in the microscope. To simulate blur, the test confocal image is convolved with a horizontal motion blur kernel (material and methods). A sample image before and after blurring is shown in Fig 8A.

The 10 test stacks were subjected to the blurring function and were segmented using the 5 pipelines (Fig 8E). VJI values were then computed for each of the results and plotted (Fig 8B). It is seen that the Plantseg, MARS, and CellPose pipelines are relatively less affected by blurring, whereas this effect produces larger variability in results in the UNet +WS pipeline. The effects on rates of undersegmentations remain relatively low (Fig 8C). Apart from Plantseg and MARS, the other pipelines suffer higher rates of undersegmentation (Fig 8D) under the impacts of blurring.

**Fig 9:**
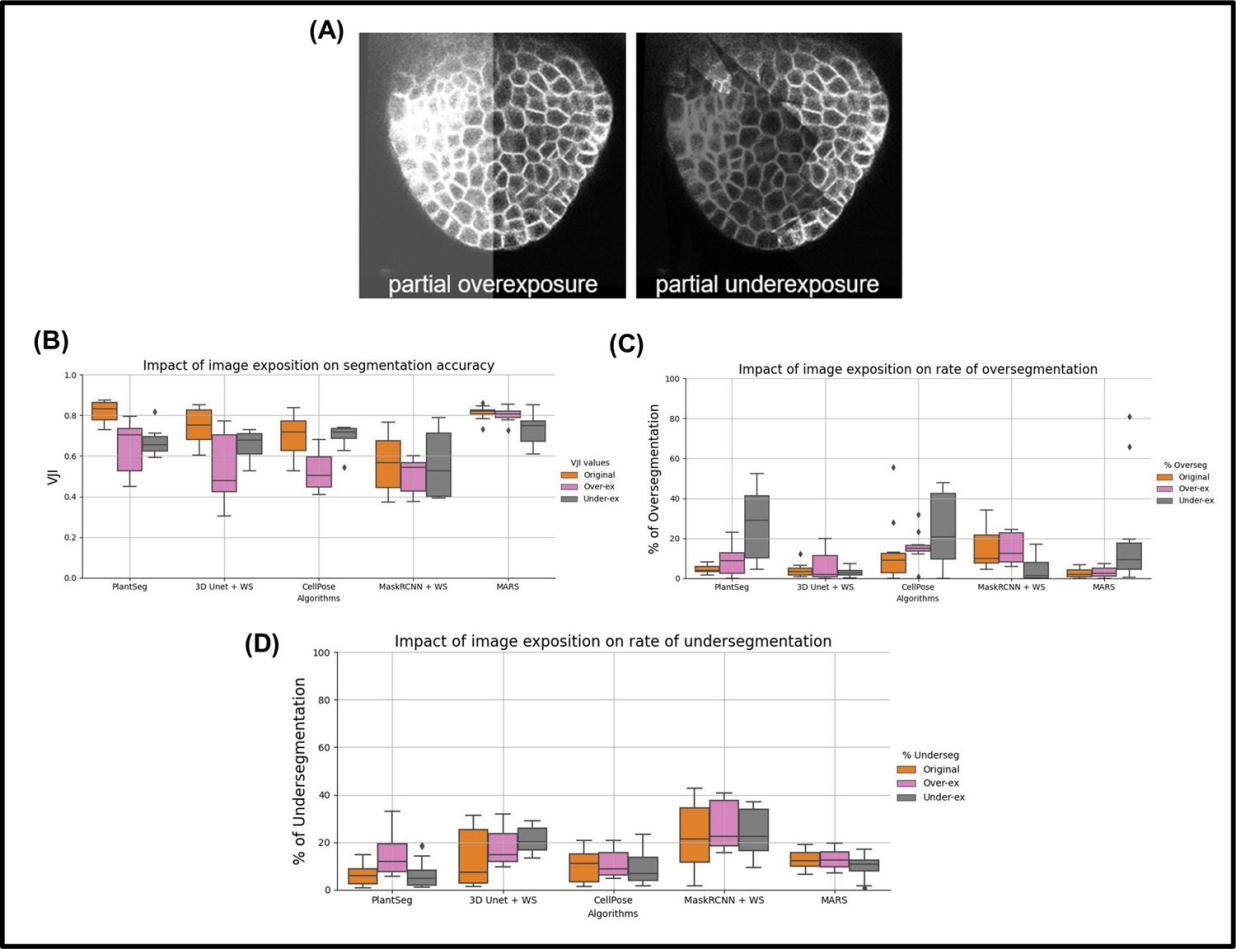
Impact of image exposure levels on segmentation quality of 5 pipelines. **(A)** Examples of partial over- and underexposure. In **(B)**, the VJI values for over- and underexposure are plotted together with the original VJI values for unmodified stacks. Similarly in **(C)** and **(D)**, the rates of over- and undersegmentation are plotted for the impacts of over- and underexposure alongside those for the unmodified stacks. VJI, volume-averaged Jaccard index.

**Fig 10:**
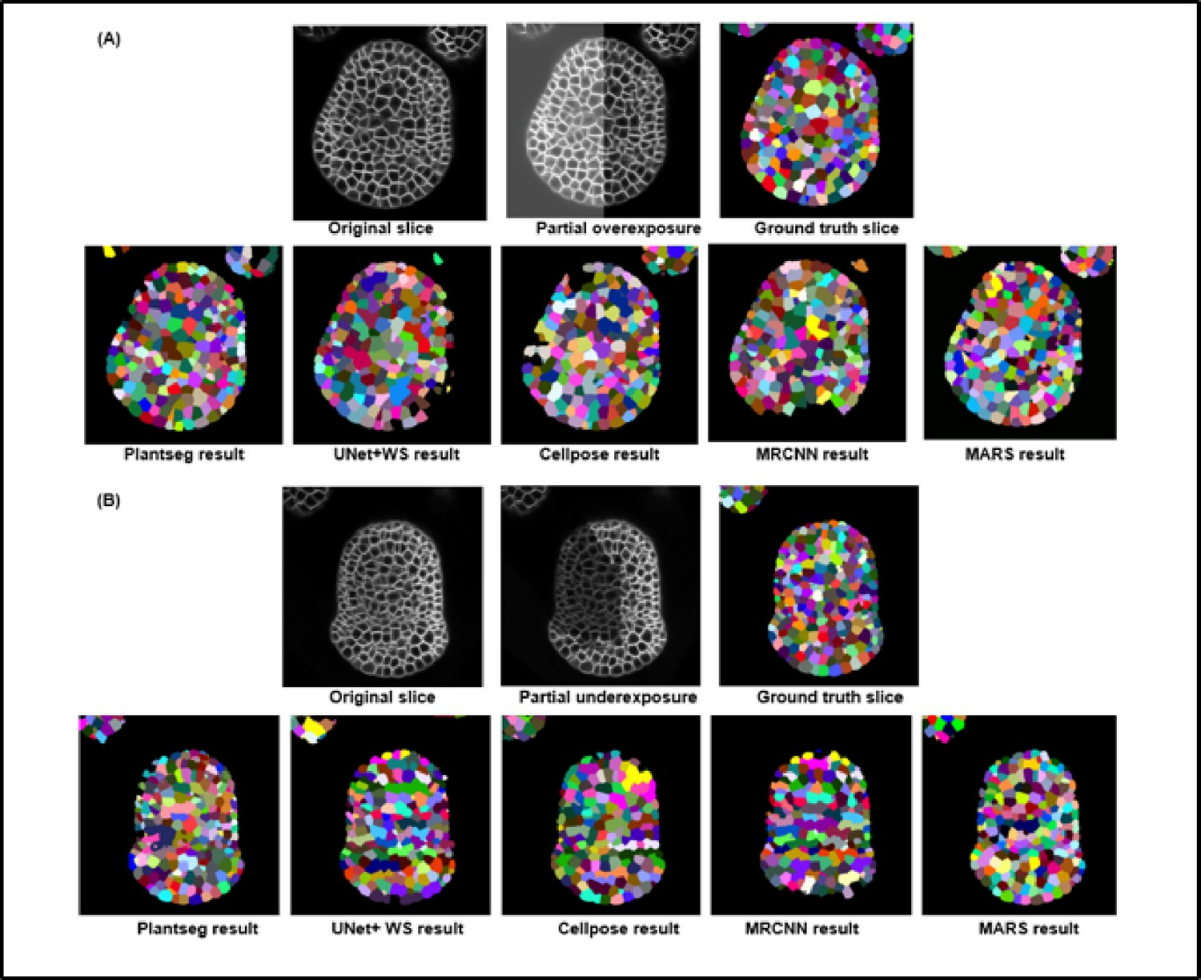
**(A)** Sample results from the 5 pipelines under the impact of image overexposure. **(B)** Results from the 5 pipelines under the impact of partial underexposure.

**Image intensity variations.** Partially bright regions in microscopy images may be caused by inhomogeneous illumination sources and shadow effects are mostly caused by presence of light absorbing objects or obstructions [39,40] or due to a nonhomogenous cell membrane marker. To emulate the effect of such intensity variations within an image, partial overexposure or underexposure regions (Fig 9A) were imposed on the test images, which were then segmented using the 5 pipelines. The VJI values and rates of over- and undersegmentations for all the results were computed and are shown in the box plots for all stacks in Fig 9B–9D. Sample results from all pipelines for overexposure are shown in Fig 10A and for underexposure in Fig 10B.

**Overexposure** had a strong negative impact on the VJI of Plantseg, UNet+Watershed, and Cellpose. The MARS and Mask R-CNN pipelines were not affected appreciably. Oversegmentation increased significantly in the Plantseg and Cellpose (Fig 9C) more than other pipelines. Undersegmentation (Fig 9D) was higher in PlantSeg and UNet+WS, while for others, it remained at similar levels as on original images.

**Underexposure** was strongly reflected in the VJI results from Plantseg and also on MARS (Fig 9D). Partial underexposure induced a high degree of oversegmentation in MARS, PlantSeg, and CellPose and high rates of undersegmentation in the UNet+WS pipeline. The Mask R-CNN–based pipeline, although having low overall accuracy, was found to be less sensitive to underexposure.

In conclusion, overall, for the Plantseg, UNet+Watershed, and Cellpose (or the UNet based) pipelines, the effect of image intensity variations appears to be much stronger than image noise or blur effects. Mask R-CNN and, to a lesser extent, MARS, on the other hand, were relatively stable. MARS out of all the pipelines is found to be the most stable under the effect of image artifacts, although it leads to increased oversegmentation in partially underexposed samples.

#### Strategy 4: Evaluating pipelines on unseen data types

We tested the performance of our trained DL pipelines and MARS on data different from confocal images of floral meristems, for example, on data from other microscopes and tissue types to observe the adaptability of our methods to new and unseen data (the DL pipelines were originally trained on SAM images). Images from 2 datasets were used for this and the results from all the pipelines are presented below.

**(a) Ascidian *Phallusia mammillata* (PM) embryo images.** The 5 pipelines were used for segmenting 3 images from the PM embryo dataset, described in [41]. These were captured using multi-view Light-sheet (MuVi-SPIM) microscopes from fluorescently labeled cell membranes. Ground truth segmentations for these images were also provided in the dataset.

**Fig 11:**
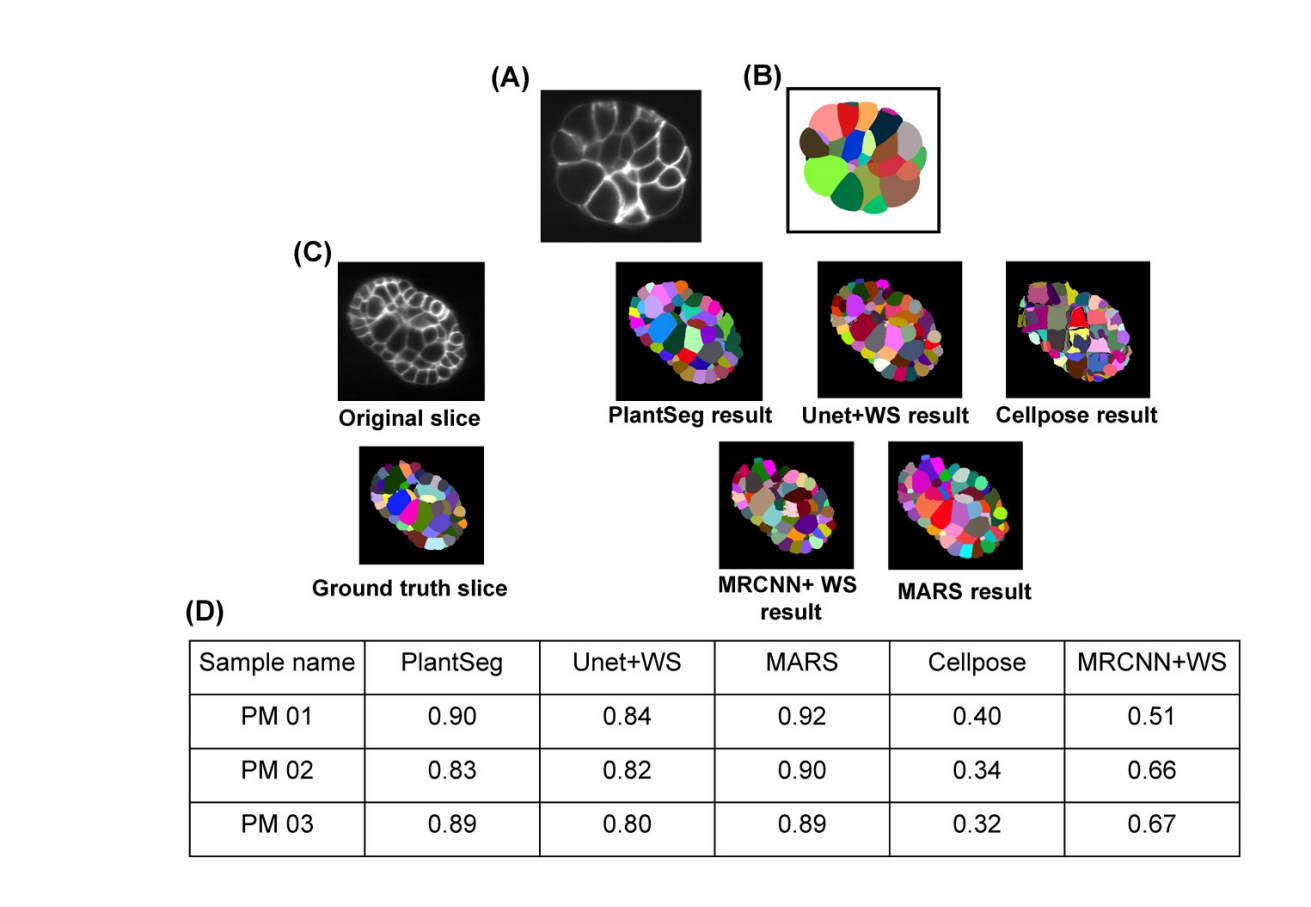
Slice view of a sample **(A)** Ascidian embryo image and its **(B)** ground truth segmentation. **(C)** Ascidian embryo image (PM03), ground truth, and segmentations by 5 pipelines. **(D)** VJI values for segmentation results using Ascidian PM data and 5 pipelines. PM, *Phallusia mammillata*; VJI, volume-averaged Jaccard index.

Three test images and their ground truths were taken from each of the above datasets, and all 5 pipelines are used to segment this data. Then, the VJI metric was used to estimate the segmentation quality as done with the floral meristem test dataset (Fig 11D).

The DL pipelines Plantseg and UNet+WS provide high accuracy results on completely unseen data without requirement of retraining (Fig 11D). The MARS algorithm after slight tuning of the parameters (mainly image smoothing sigma) for this dataset provides the highest accuracy segmentations. The MRCNN+WS results are similar to what we got previously from this pipeline on floral meristem data. The Cellpose accuracy however falls from their average values observed for floral meristem data.

**Fig 12:**
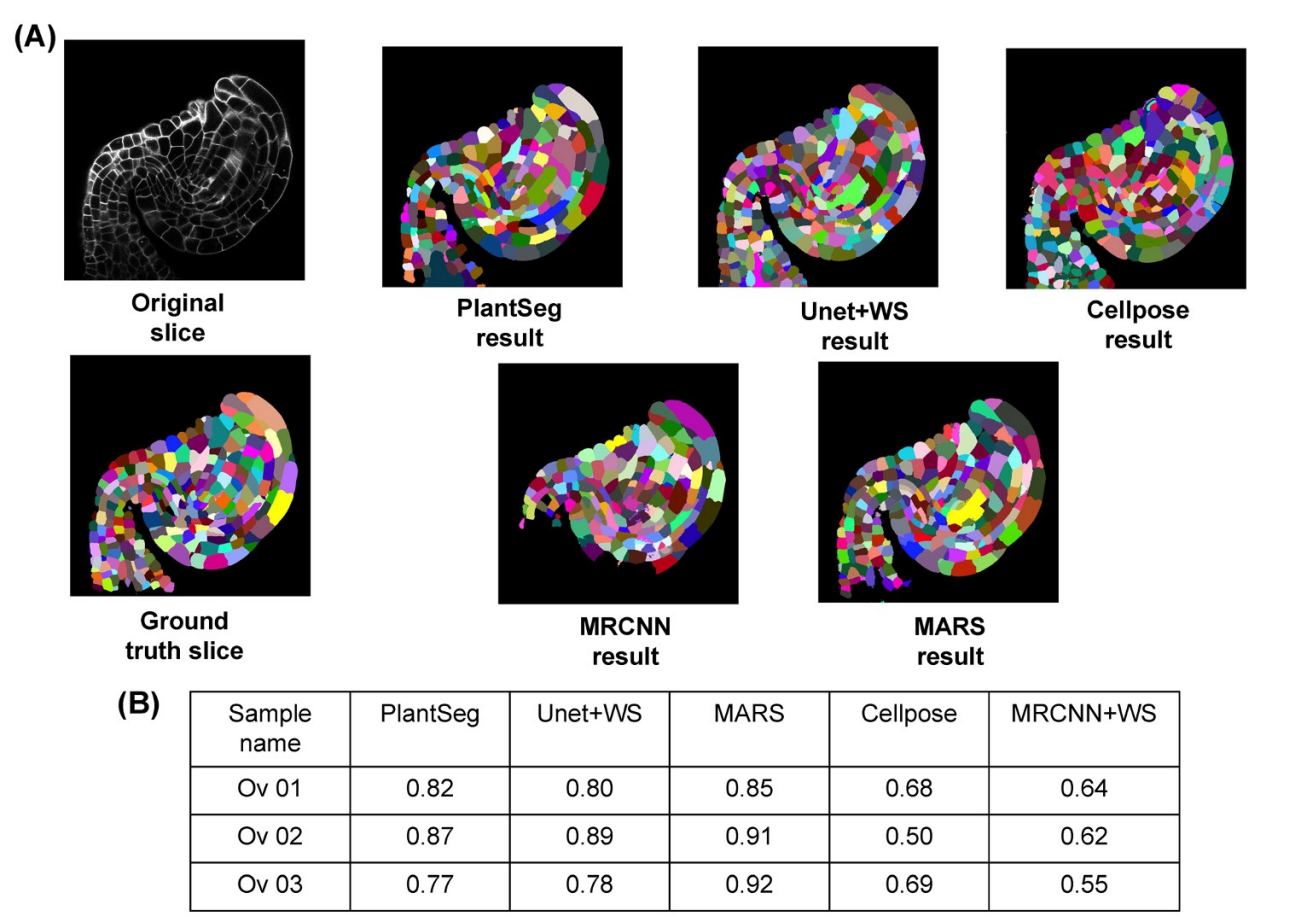
**(A)** Ovule image and ground truth along with segmentations by 5 pipelines. **(B)** VJI values for segmentation results using ovule data and 5 pipelines.

**(b) *Arabidopsis* ovule images.** Three-dimensional confocal Images of *Arabidopsis thaliana* ovules at various developmental stages were taken from the dataset provided by the authors of the Plantseg pipeline [26]. Three stacks from this dataset along with their ground truths were used for evaluating the 5 pipelines. Structurally, these are quite different from *Arabidopsis* floral meristems, and the segmentation results from the 5 pipelines along with the original and ground truth images are shown in Fig 12A. Results from the evaluation are in Fig 12B. Note that MARS needed extensive retuning for this dataset (discussed further in the Materials and methods section).

### Three-dimensional visualization of segmentation quality

At present, visualization and exploration of 3D image data are possible by software packages such as ImageJ, ParaView, or MorphoGraphX [42]. However, only a few tools allow users to project any extrinsic property over an image and to interact with a 3D image dataset at cellular resolution. One such platform is Morphonet [43] (see S2 File), which is an open-source and web-based platform for interactive visualization of 3D morphodynamic datasets. These datasets are created by converting image datasets (3D stacks) into corresponding 3D meshes. So far, Morphonet has been used to project a variety of genetic and morphological information such as gene expression patterns, cell growth rates, and anisotropy values on plant and animal tissue images.

**Fig 13:**
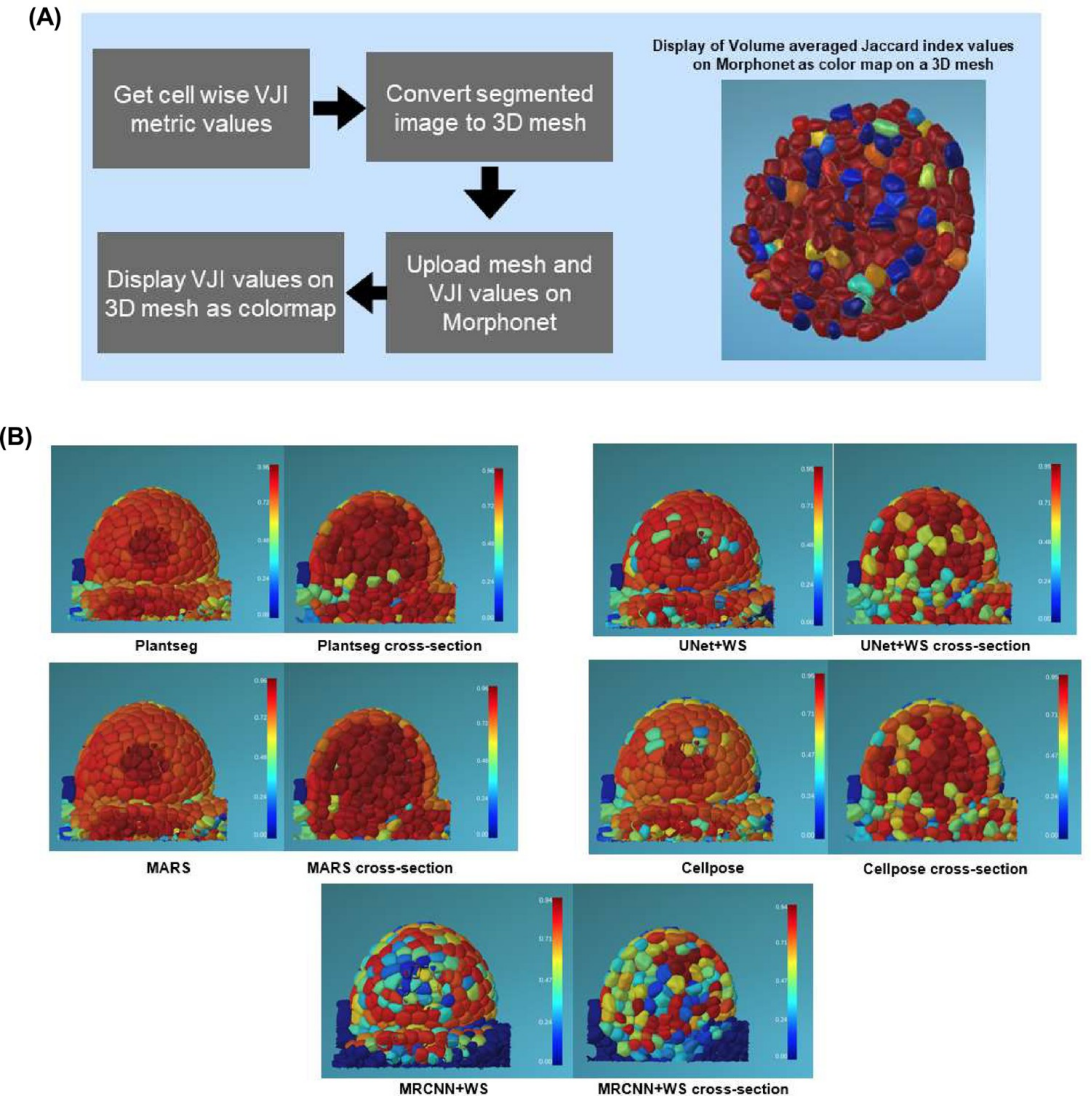
**(A)** Process to view segmentation quality in 3D on Morphonet.Segmentation quality results (VJI values) for a test stack (TS2-26h) from 5 pipelines displayed on Morphonet. Users can slice through each 3D stack in XYZ directions and check the property (here VJI values) for each cell in the interior layers of the tissue structure. For example, for each pipeline in the above figure, the left image shows the full 3D stack, and the right image shows the cross section of the same stack after slicing 50% in the Z direction. VJI values are projected as a “property” or color map on the cells. In this figure, a “jet” color map is used where red represents high, and blue represents low VJI values as shown in the color bars alongside.3D, three-dimensional; VJI, volume-averaged Jaccard index.

Here, we have developed a MophoNet-based method for interactive 3D visualization of segmentation quality measures, by projecting VJI values on 3D meshes created from 3D confocal images (Fig 13). The 3D meshes were created by the marching cubes algorithm [44] and uploaded on MorphoNet. The VJI values for the image were computed and uploaded as a quantitative property of each individual 3D cell of the image for each segmentation pipeline. The VJI values were then viewed using color maps on the Morphonet browser.

The steps of the Morphonet-based visualization pipeline are provided as open resources in the SegCompare Gitlab repository (described in S1 File) so that users can upload their own segmentation results on our datasets from any segmentation method or pipeline and benchmark study their accuracy characteristics. The only requirement is that these segmentations should be 16-bit labeled.tif files and must have their ground truth segmentations for evaluation. With segmented and ground truth.tif stacks, the steps for Morphonet-based segmentation quality visualization include (see Fig 13A) (a) estimation of cell-by-cell VJI and converting this information to Morphonet compatible format; (b) mesh calculation from the ground truth segmented image and converting it to Morphonet compatible.obj format; and, finall,y (c) logging in to Morphonet, creating a dataset, and uploading the mesh and VJI information. All these steps are documented and implemented in an easy to use Python notebook as described in S1 and S3 Files.

The benefits of the Morphonet-based visualization is that a user can (a) check the VJI value for each cell by clicking on it; (b) impose a color mapping of the Jaccard index values so that users can at a glance observe the overall distribution of the VJI values on the cells of the 3D stack (Fig 13B) as well as locate cell regions where poor Jaccard values are found or concentrated; (c) interact with segmented data in 3D, e.g., rotate 360 degrees or zoom into cells or slice through the segmented data in XYZ directions and inspect segmentation quality in any inner cell layers; and (d) requires no special software or hardware installation. Also, data uploaded on Morphonet can be shared with multiple users who can directly access it on the Morphonet platform.

Examples of viewing segmentation quality on Morphonet is illustrated in Fig 13B where mesh belonging to a test stack is uploaded on Morphonet and the VJI values for all the segmentation pipelines are projected on it individually to observe the cell-by-cell variation in segmentation quality.

Sample segmentation accuracy data and 3D meshes in Morphonet compatible format are provided in our repository: https://figshare.com/projects/3D_segmentation_and_evaluation/101120.

Sample videos demonstrating various aspects of the 3D visualization process on Morphonet may also be found under “Videos” in our repository described in S3 File: https://doi.org/10.6084/m9.figshare.14686872.v1.

## Discussion

In this study, we developed a benchmarking procedure for evaluating 3D segmentation pipelines and used it to analyze 4 DL segmentation pipelines and a non-DL one. Initially, it was difficult to predict the relative performance of the individual pipelines, because they were applied on diverse datasets and used different evaluation metrics. Most common evaluation strategies consist of estimating simple numerical metric values, which also vary widely between the methods. For example, in the Plantseg original paper [26], the variation of information metric is used, while other studies are based on the Jaccard index, such as the SEG score in [34,31] or the aggregated Jaccard index metric (AJI) in the original UNet+WS paper [24]. The numerical results from these different metrics are difficult to compare since they are based on different concepts and/or use different datasets. We have used here the VJI. Like the SEG metric defined in [34,35], it is an average Jaccard index of all pairs of compared regions, but takes into account the cell volume, thus reflecting the error per volume, rather than per cell. Therefore, the VJI is more adapted to our data, where cell size can vary considerably.

Another problem is that the simple segmentation accuracy estimates do not provide a detailed view about the types of segmentation errors present in the segmentation results or how the errors are spatially distributed. Therefore, in addition to the VJI metric, we evaluated the segmentations by measuring the rates of over- and undersegmentations, which indicates the strengths and weaknesses of specific segmentation procedures. This spatial distribution of segmentation quality was further addressed through a layer-wise evaluation in this paper. The range of metrics presented here provided an in-depth analysis of the type and magnitude of the errors as well as their spatial distributions produced. Each of the metrics gives its own specific information, which finally allowed us to define the advantages and shortcomings of each segmentation pipeline. We have also provided full details and coding implementations of the metrics used in the benchmarking process so that they can be reused by others.

The MorphoNet-based interactive evaluation is a fast and efficient way to visualize segmentation quality superimposed on 3D representations. It comprises 3 steps—calculation of cell by cell VJI values for a pair of stacks, creating a 3D mesh, and uploading these on Morphonet using their Python API (S2 File). All these steps are covered in 2 Python notebooks provided in the SegCompare Gitlab repository (S1 File). With this visualization, users can navigate through 3D objects in an image and study the segmentation quality for different 3D sections by slicing through the image data (converted to 3D meshes). Since the Morphonet visualization is browser based, it does not require any software installation or special hardware for 3D data visualization. This technique may therefore be added as a final step to any segmentation pipeline, DL or non DL, for a 1-step analysis of the 3D segmentation performance.

We also tried to evaluate how much better the DL algorithms performed compared to the non-DL algorithms, which is a recurrent question in DL and computer vision research. Results of PlantSeg, the best performing DL pipeline of those tested here, were matched by those of the non-DL MARS algorithm. However, without tuning, the MARS accuracy may degrade significantly over completely different datasets as observed with the segmentations of ovule images (see Materials and methods section). By contrast, DL models, especially PlantSeg, once trained, performed well throughout. With regard to the time for execution, the MARS segmentation would take much longer than the PlantSeg pipeline (25 to 30 minutes for an individual stack versus 8 minutes, respectively; see Material and methods section).

The quantitative impact of image artifacts on the accuracies of segmentation pipelines are not always analyzed in literature. This is why one of our protocol strategies was to estimate the impact of image artifacts like blur, over-, and underexposure and 3 levels of Gaussian noise on the VJI values and rates of over- and undersegmentation of the 5 segmentation pipelines. The size of our test dataset therefore consisted of a total of 60 3D stacks with 10 original and 50 synthetically modified ones. In addition, completely unseen 3D images from plant and animal datasets were also segmented using our pipelines.

An important question is why the pipelines have different levels of performance. This could be attributed firstly to the construction of the pipelines. We observed that, although 2D segmentation pipelines may be adapted to perform 3D segmentations (e.g., as done in Cellpose and MRCNN+WS), the end-to-end direct 3D segmentation pipelines (such as Plantseg and UNet+WS pipelines) achieved higher accuracies. This trend is observed in the segmentation accuracy results of the pipelines for 10 original stacks as well as with the stacks having artifacts. Another possible reason behind the difference in performance, especially between the 2 end-to-end 3D pipelines (Plantseg and 3D UNet+WS), could be arising out of the postprocessing strategies used in them. Plantseg provides 2 semantic output classes (background and boundaries), while 3D UNet+WS produces 3 classes (background, boundary, and cell interiors). For the final instance segmentation, Plantseg relies on graph partitioning, while UNet+WS uses watershed. It may also be noted that the 2 pipelines Plantseg and UNet+WS are designed specifically for boundary detection of fluorescently labeled cell boundaries, which matches the type of data that we have used in our work. Cellpose, on the other hand, is built for segmenting a wide range of bio-image data types such as cytoplasm, membrane, and cells without fluorescent markers. While the UNets of Plantseg and UNet+WS pipeline extracts and operates on the cell boundary images, the Cellpose pipeline involves prediction of spatial gradient features and a cell probability map that determines the inside and outside regions of cells. The Mask R-CNN does not operate on the boundary prediction principle at all but uses object detection and classification for prediction of segmented regions. Thus, the difference in performance of the 4 DL algorithms could be attributed to their DL model architectures, the way the full segmentation pipeline is constructed as well as on the type of data they are applied to.

We also observed that responses of DL pipelines are different to training data augmentation (see S5 File). The performance of the Plantseg pipeline improves on adding images with artifacts (over- and underexposed images) in the training set. By contrast, the performance of the UNet+WS pipeline does not generalize well after using the overexposure augmentation as its performance degrades on normal images. Its performance, however, improves overall with the underexposure augmentation. We therefore found that same augmentations may not necessarily yield better results for all DL-based segmentation pipelines. It would also be of interest to investigate the performance of these DL models if different types of image data (e.g., membrane stained, nuclei stained images, images from other tissue types, etc.) are mixed in the benchmark training set.

### Data and code availability

All the data and coding implementations from this work are available as open resources. The methods for reproduction of the segmentation pipelines and the segmentation evaluation techniques are documented in the Gitlab repository named SegCompare (https://gitlab.inria.fr/mosaic/publications/seg_compare) along with relevant resources in Jupyter notebooks. The 3D confocal training and test datasets used in this work are provided in open data repositories. These are described in detail in S1 and S3 Files.

## Materials and methods

### Training data

**Fig 14:**
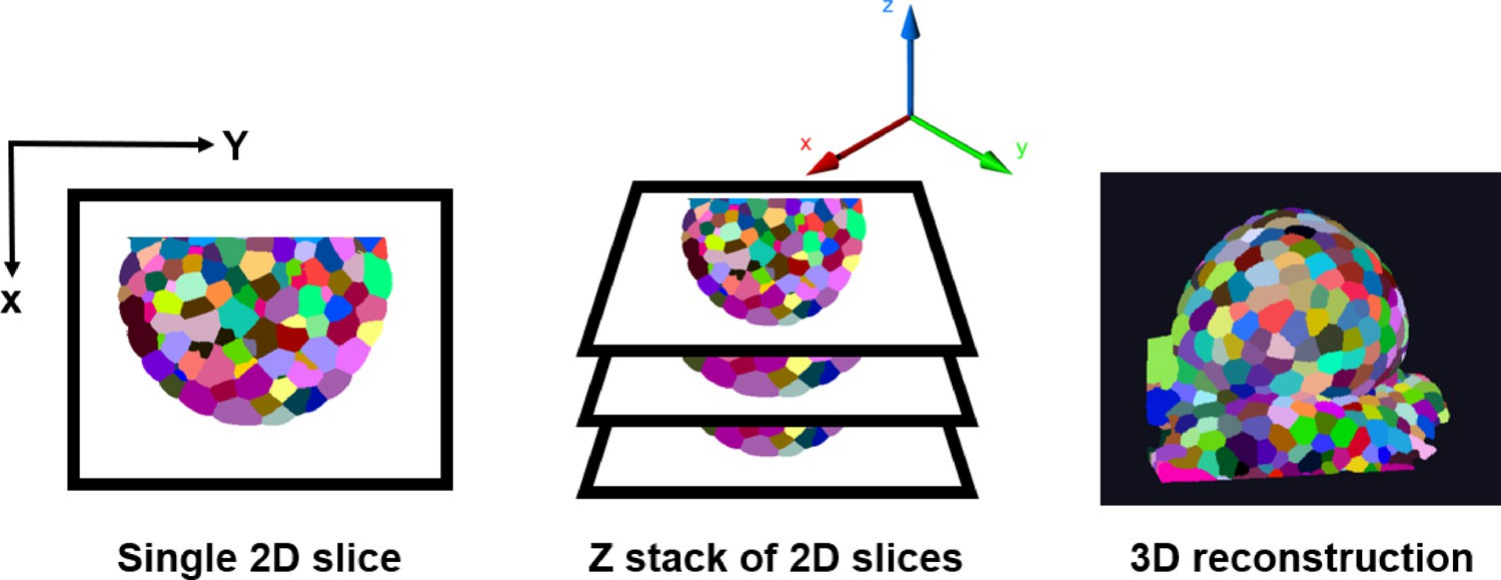
A confocal image is made up by scanning through each point on a 2D plane of an object. The 3D confocal image is made up of such 2D frames stacked along the Z-axis. Using the 2D Z slices, a full 3D view of the object can be reconstructed. 2D, two-dimensional; 3D, two-dimensional.

The training data for all the DL algorithms comprise 3D confocal image stacks and their corresponding ground truth segmentations. The structure of a 3D confocal image is shown in Fig 14.

**Fig 15:**
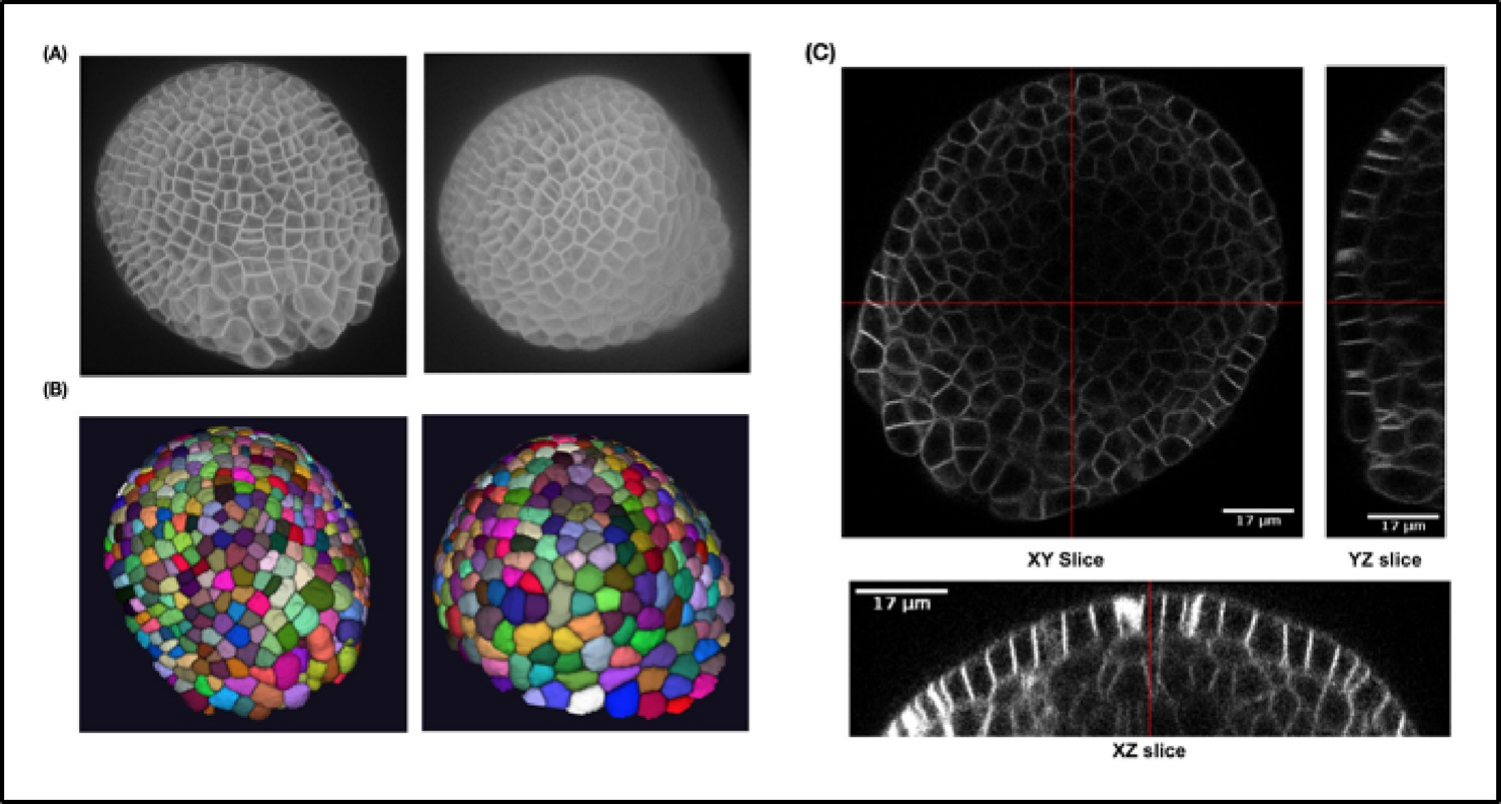
**(A)** Three-dimensional projection of 2 training images and **(B)** corresponding ground truth segmentations. **(C)** Lateral (XY) and axial slices (XZ and YZ) of a sample confocal training image.

The source of the training data is *Arabidopsis thaliana* SAM, which is a multicellular tissue (Fig 15A and 15C) where cells at the surface grow radially outward from the central to the peripheral zone. These data have been previously used and published in [36]. The SAM 3D stacks were captured using a time-lapse confocal microscope for every 4 hours for 0 to approximately 80 hours and were passed through a pipeline of image correction steps. The mean size of the stacks is 150 × 512 × x × 512 pixels (zyx dimensions).

For each stack, slice misalignments due to vibrations or microscope stage movements were corrected via translation transformations using the StackReg module of ImageJ. Also, z-slices with horizontal shifts were replaced with the closest z-slice with no shifts. Imaging errors during vertical movement of the plant due to growth were also compensated for by estimating stretching constants for each stack by comparing rapidly acquired low-z-resolution stacks with slowly acquired high-z-resolution stacks (S10 Table in SI Appendix of [36]). In addition, Gaussian and an alternative-sequential filtering was done for noise removal.

The ground truth data for training (Fig 15B) consists of the 3D segmentations of the above image stacks done by 3D watershed followed by extensive manual corrections on a slice-by-slice basis. For corrections of segmentation errors, cell boundaries were estimated from the segmented images superposed on the original images for visual inspection. For errors due to over- or undersegmentation or missing cells, the noise filter and watershed parameters were adjusted until satisfactory segmentations were obtained for peripheral as well as central zones of the SAM images. In the ground truth images, voxels, which belong to the same cell, have the same label.

### Test dataset

For testing the segmentation algorithms, a dataset of ten 3D confocal image stacks was used (described in [45]).Test Set 1 (TS1) contains 6 stacks (0-hour, 24-hour, 32-hour, 72-hour, 120-hour, and 132-hour time points) are from one meristem (FM1 in [46]). Time points of Test Set 2 (TS2; 26 hours, 44 hours, 56 hours, and 69 hours) correspond to FM6 in Refahi and colleagues. Images of the stacks are shown in Fig 4. These images are from the floral meristem of *Arabidopsis* from initiation to stage 4. Corrections for alignment and vertical movements are described in [46]. Expert ground truth segmentations of these test stacks were also available to perform numerical comparisons between crops of the segmented results and corresponding ground truths.

To evaluate the segmentations, crop regions were defined on each test stack and corresponding ground truth image. This is done firstly to avoid low-intensity regions in the raw images, which often correspond to instances of incorrect segmentations in the ground truth images. Second, It was observed that some of the hybrid pipelines faced memory issues when handling very large volume stacks. Therefore, the crops were done such that the volumes of all raw test stacks could be processed by all pipelines. Homogeneous crops of the 10 raw images and corresponding ground truth images were therefore created and used for evaluating all the pipelines for all the experiments described in this work.

### Software and libraries used

The software/libraries used in this work include Timagetk for implementing the MARS algorithm (https://mosaic.gitlabpages.inria.fr/timagetk/index.html), Numpy [47], and Matplotlib [48] for plotting the results. Fiji/ImageJ [49] was used for image visualization. StackReg [46] was used by the creators of the training and test datasets. Tensorflow [50] and Pytorch were used for implementing the DL models.

### Training and segmentation details for DL pipelines

The 4 DL algorithms were first trained using the common meristem dataset. The details on how these pipelines could be reproduced are described in the Gitlab repository (S1 File). For training the DL networks and testing, a CUDA enabled NVIDIA Quadro P5000 GPU was used with an Intel Xeon 3 GHz processor. Python 3.x is the default programming language for training/testing all the segmentation pipelines and implementing the evaluation metrics.

#### Plantseg

**Fig 16:**
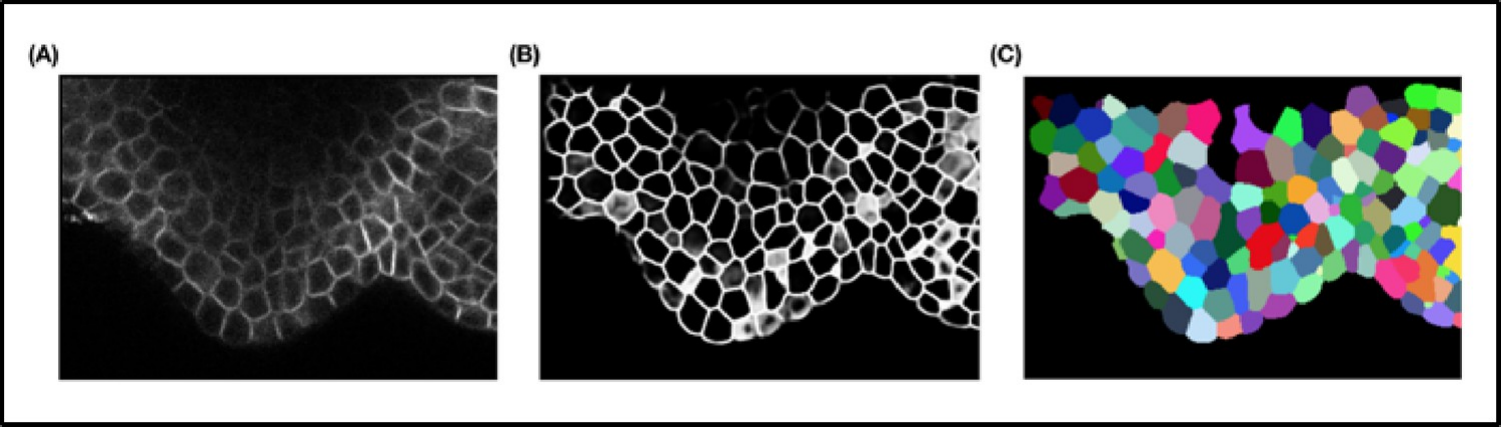
Plantseg workflow. **(A)** Input image. **(B)** Boundary prediction. **(C)** Final segmentation.

The residual 3D UNet as described in [26] was used for training on our data. The Residual 3D UNet is a variant of the 3D U-Net and comprises a contracting encoder and an expanding decoder part but uses residual skip connections in each convolutional block in the U-Net. For final segmentation of our test data, all 3 of the graph partitioning postprocessing strategies (GASP, Mutex, and Multicut) were found to provide similar results, so GASP was used to obtain all the final segmentations from Plantseg that are evaluated in this work (Fig 16).

The optimal parameters for training the 3D Residual UNet model of the Plantseg pipeline were found through experimentation, and the best parameter values are reported here. For training, the Adam optimizer was used with a learning rate of 0.0002. The loss function used was BCEWithLogitsLoss after experimenting with other loss functions like Dice, BCEDiceLoss, and BCEWithLogitsLoss. The weight decay was set to 1.0e-05. For data augmentation, random flip and rotation was used along with standardization (Z-score normalization) of the raw image. The number of training epochs required was 500 until the validation loss reduced no further. The evaluation metric used was BoundaryAdaptedRandError. The time taken per epoch is around 550 seconds. Note that Plantseg training configuration allows setting of iterations besides epochs, which was varied between 10,000 and 150,000 during the experiments.

#### UNet+Watershed

**Fig 17:**
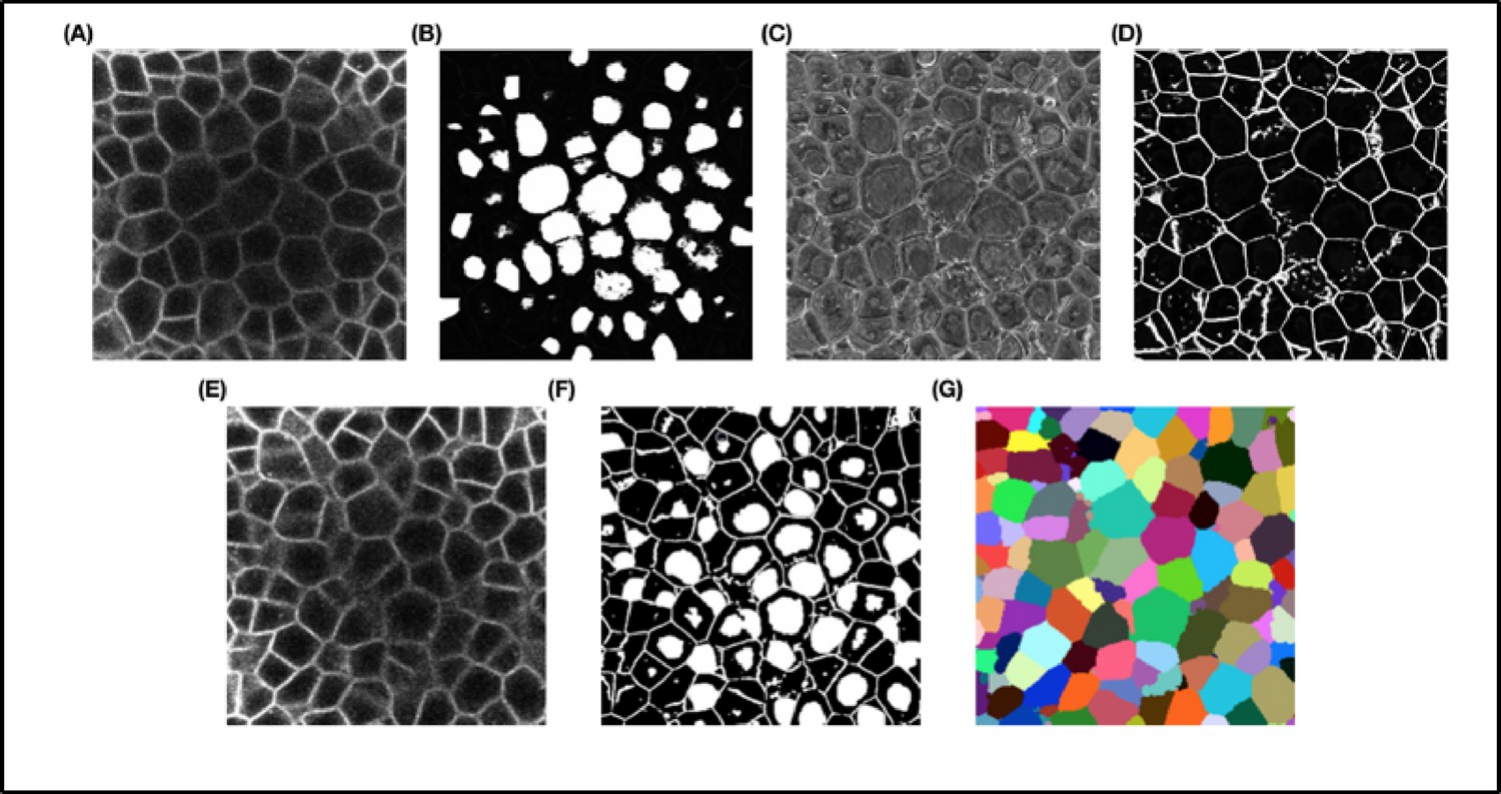
Three-dimensional UNet+ WS workflow. **(A)** An input confocal image (xy slice). **(B)** Class 0 prediction—centroids. **(C)** Class 1 prediction—background. **(D)** Class 2 outputcell membranes. **(E)** An input confocal image (xy slice). **(F)** Seed image slice. **(G)** Final segmented slice using watershed on (F).

The 3D UNet module of the pipeline was trained using the custom training dataset described above. The 3D UNet predicts 3 classes of output images from an input 3D confocal stack. These classes are cell centroids, cell membranes, and background maps (Fig 17B–17D). Dimensions of these 3 output images are the same as that of the input stack.

Using these 3 predicted image classes a seed image is obtained by first thresholding the centroid maps (0.8 times the max intensity), followed by its morphological opening (circular kernel, size 5 × 5 × 5) and subtracting the membrane and background maps from the resultant image. An example seed image is shown in Fig 17F, and the corresponding seeded watershed segmentation output is shown in Fig 17G.

The optimal parameters for the 3D UNet model were found through trials with different numbers of epochs (400 and 800), variation of the type of loss function used (mean-squared error and custom loss), and batch size variations of 5 to 20. The optimal number of epochs were found to be 400, and a batch size of 5 was used. The evaluation metric used was mean intersection over union or MIoU. The custom loss function used during training is called weighted_binary_class_crossentropy and is a binary cross entropy–based loss with class weighting (0.3 for each of 3 output classes). The optimizer was Adam, and a learning rate of 0.001 was used along with batch normalization. For data augmentation, flipping and rotation was used. The UNet watershed pipeline as proposed by the authors does not include any data normalization function in their training configuration. Batch size is set to the default value of 5. The time taken per epoch is around 750 seconds.

#### Cellpose

The Cellpose pipeline uses a 2D UNet to predict horizontal (X) and vertical (Y) flows along with a pixel probability map for each 2D test image. Using the XY intensity gradients or vector fields, pixels belonging to each object to be segmented can be aggregated around the centroid region for that object. For the final segmentation in 3D of the test sets, Cellpose uses the 2D trained model to predict the horizontal and vertical gradients for each of the XY, XZ, and YZ sections of a 3D volume. These 6 predicted gradients are then averaged pairwise to obtain the final XYZ vector map in 3D.

The 2DUNet module was trained using 2D slices (512 × 512 pixels) from the custom training dataset along with their corresponding 2D masks. The tunable parameters of the method such as flow_threshold, cell probability threshold, and cell diameter were set to the default values of 0.4, 0.0, and 30 pixels, respectively.

Optimal values of different model parameters used for the Cellpose training included batch_size = 8, diameter = 30, learning_rate = 0.2, weight_decay = 0.00001, and momentum = 0.9. Rotation and flipping were used as augmentations. For data normalization, the authors of the pipeline use a custom function that normalizes images so that 0.0 is first percentile and 1.0 is 99th percentile of image intensities. The same function was used by us. The model used a SGD optimizer and a SigmoidBinaryCrossEntropyLoss loss function and the training took 2,050 epochs. The learning rate hyperparameter was tuned for this model between 0.0004 and 0.2 to observe the results. The best results were obtained for a learning rate of 0.0008. The time taken per epoch is around 600 seconds.

#### Mask RCNN

Mask R-CNN (also called MRCNN in this paper) uses a backbone network such as Resnet-50 or Resnet-101 to extract image features followed by generating region proposals of objects (to be segmented) using a RPN. These region proposals are refined and fed to a fully convolutional classifier to identify object classes. The final output of MRCNN includes (1) boundary box for each object instance; (2) pixel level mask for each object identified; (3) class predictions for each object instance; and (4) confidence score of each prediction.

**Fig 18:**
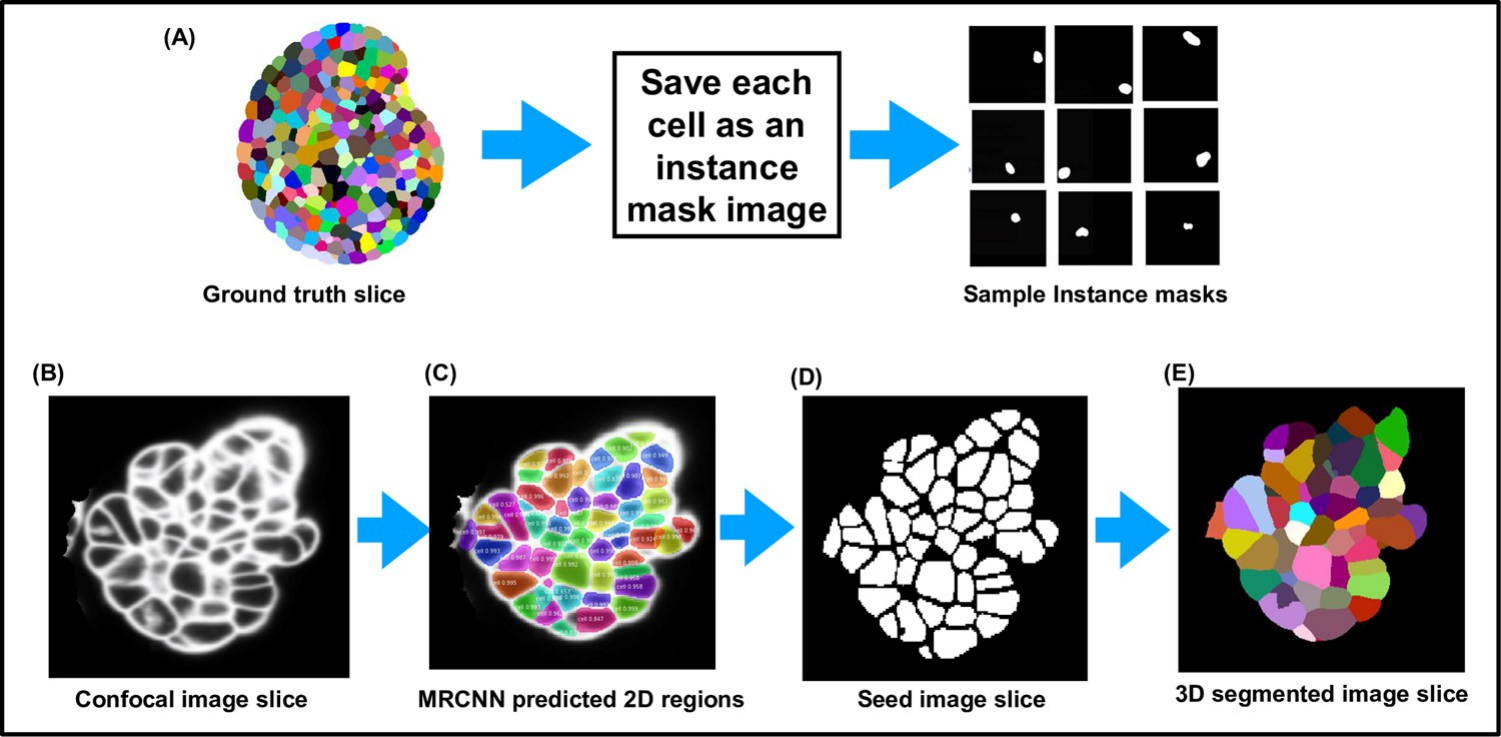
MRCNN+Watershed workflow. **(A)** Creation of instance masks for training MRCNN. **(B)** Example confocal slice. **(C)** Two-dimensional predictions by MRCNN. **(D)** Binary seed image created from identified cell regions in (C). **(E)** Same slice after 3D segmentation using watershed on the binary seed image. 3D, three-dimensional.

In the MRCNN with watershed pipeline, using a 2D trained Mask R-CNN model, the cell regions in each Z slice of a 3D volume are predicted (Fig 18C). The predicted Z slices with the identified cell regions are stacked together to produce a 3D binary seed image, which is then labeled using a 26-neighbor connected components labeling method. The labeled seed image is then used for watershed-based postprocessing to obtain the final 3D instance segmentation. The Mask R-CNN algorithm with a Resnet 101 backbone network was trained using 2D images along with instance masks for each object (to be segmented) in the image. Using Resnet 50 as a backbone network did not give satisfactory results. Training images (2D slices) from the custom training dataset were used, and the instance masks were generated from the corresponding ground truth 2D masks (each labeled cell region forms an instance mask) as shown in Fig 18A. Both the raw image and the set of instance masks for each image are then used for training the Mask R-CNN network.

The Mask R-CNN model provides a range of hyperparameters to tune. For training the Mask R-CNN model in this work, the parameters like RPN threshold, RPN anchor scales, number of anchors per image, number of training ROI’s per image, etc., were varied and tested through experiments. The optimal values for these were found as follows: RPN threshold = 0.9, RPN anchor scales = (8, 16, 32, 64, and 128), number of anchors per image, number of training ROI’s per image = 300, and detection max instances = 400. An SGD optimizer was used with a learning rate of 0.001, momentum of 0.9, and weight decay of 0.0001. The loss function used was Binary cross entropy. Other loss functions are also defined for this model such as rpn_class_loss (RPN anchor classifier loss), rpn_bbox_loss (RPN bounding box loss), mrcnn_class_loss (loss for the classifier head), and mrcnn_bbox_loss (Loss for Mask R-CNN bounding box refinement) values were also monitored during training. The data augmentations used include random flipping and rotation of the images, and since no normalization function is defined in the original configuration, we followed the same. The time taken per epoch is around 600 seconds.

**Fig 19:**
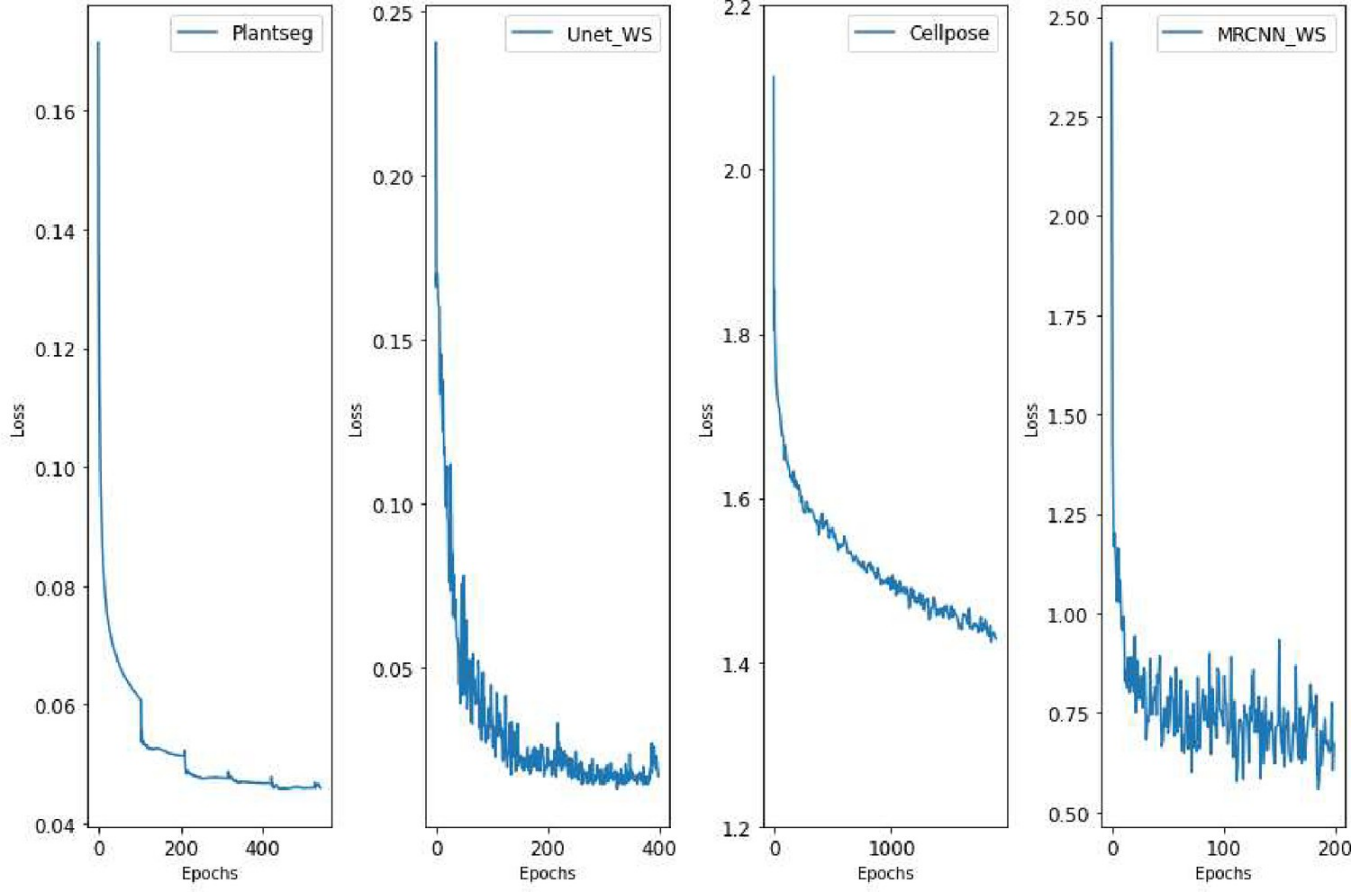
Loss versus epoch plots for training the models from 4 pipelines.

The training loss curves obtained at the end of training each of the models from the 4 DL pipelines are plotted in Fig 19. These show the optimal number of training epochs required for the models of the 4 pipelines (while training on normal images). The training time for the DL models in each pipeline are given in Table 2. The parameters to tune for each pipeline is summarized in Table 3.

**Table 2:** Training details for all DL pipelines.

Pipeline	Deep model	Optimizer	Loss function(s)	Epochs	Training time	Augmentations
Plantseg	3D Residual UNet	Adam	BCE with logits loss	500	76 hours	Rotate, flip, elastic deformation
3D UNet+WS	3D UNet	Adam	Weighted binary class cross entropy	400	83 hours	Rotate, flip
Cellpose	2D UNet	SGD	Sigmoid Binary Cross Entropy	2,050	34 hours	Rotate, flip
MRCNN+WS	Mask R-CNN	SGD	Binary cross entropy	200	33 hours	Rotate, flip

3D, three-dimensional.

**Table 3:** Parameters to tune for each segmentation pipeline.

Pipelines	Parameters to tune
Plantseg	Preprocessing: Interpolation (up to degree 2), Gaussian, or median filter Postprocessing: CNN predictions threshold, watershed seeds sigma, watershed boundary sigma, superpixels minimum size (voxels), and cell minimum size (voxels)
3D UNet + Watershed	Preprocessing: None Postprocessing: Morphological opening/erosion kernel sizes for estimating watershed seeds from UNet outputs
Cellpose	Preprocessing: None Postprocessing: Cell diameter, cell probability threshold, and flow error threshold
MRCNN+ Watershed	Preprocessing: None Postprocessing: Morphological opening/erosion kernel sizes for estimating watershed seeds from MRCNN outputs
MARS	h-minima and Gaussian smoothing sigma for image

#### MARS

No training is required for MARS, but it can require adjusting of its parameters to obtain the best segmentation results. These include the h-minima value and Gaussian smoothing sigma for the images (Table 3). We made an estimate of the execution time of the MARS algorithm on one of our sample test stacks (TS1-132h, volume = 700 × 700pixels × 297 z slices) and a single run of the algorithm takes 78 seconds on a CPU (measured from the start to finish of the algorithm execution). The parameters to tune include h-minima (integer values starting from 1, upper limit depending on image intensities) and gaussian_sigma (fractional float values varying between 0 and 1) for image smoothing. The initial setting of the parameter values and expertise of the user influences a lot the number of times these parameters need to be adjusted. So for a normal user, trial runs with 10 step changes of gaussian sigma and 10 of the h-minima parameter required a total of around 30 minutes. Additionally, manual supervision is required for inspecting the quality of segmentation in each slice of the stack, which could be estimated to take around 1 to 2 minutes after every run of the algorithm. Thus, the overall time required by MARS for segmenting a large stack like the one used above could reach up to 45 minutes. On the other hand, Plantseg (using a trained model) took 8 minutes for segmenting the same stack on a GPU-based computer and did not require any parameter tuning during this phase.

**Fig 20:**
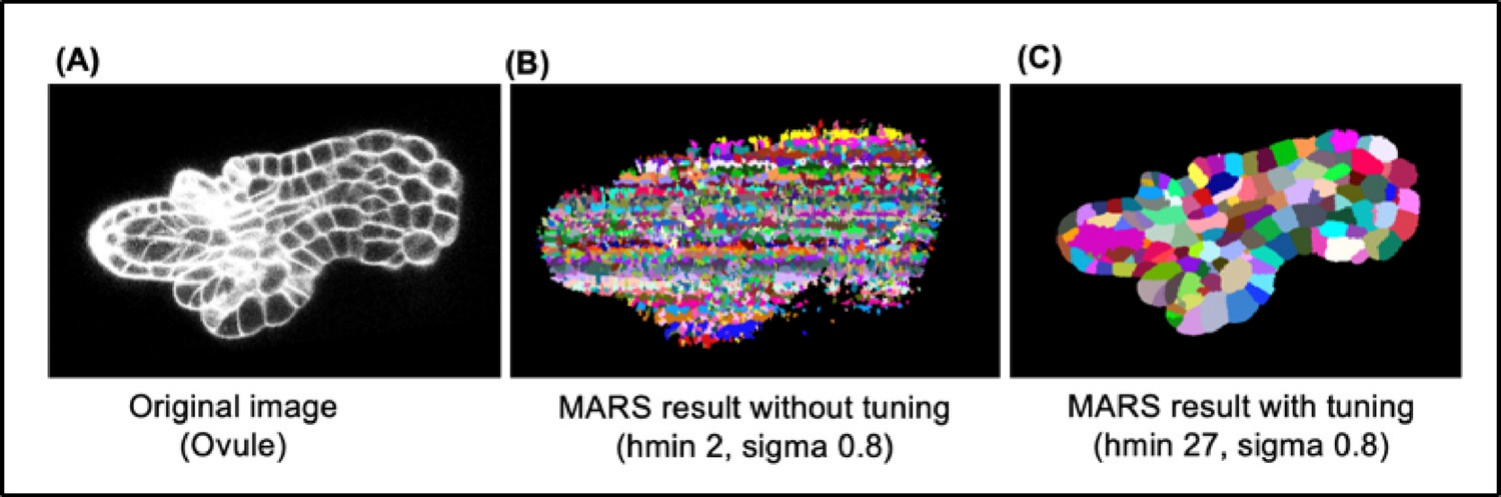
**(A)** Original ovule image. **(B)** Impact of using hmin = 2 and sigma value = 0.8 for MARS. **(C)** Result of MARS on the same image after tuning parameters.

Requirements of parameter tuning for MARS vary from one dataset to another and may be necessary for stacks within the same dataset. For example, MARS parameters used to segment our TS1 and TS2 stacks were hmin = 2 and sigma = 0.4. However, on another dataset (ovule images; see section Results, Strategy 4: Evaluating pipelines on unseen data types), a lot of tuning was necessary (Fig 20). For this dataset, we had to explore hmin values between 2 and 30 and sigma values in the range 0.2 to 0.8. Overall, more than a 100 combinations of hmin and sigma parameters had to be tested to identify the best one that yields the highest VJI. The best accuracy (given in Fig 12B) was obtained for hmin values of 27, 27, and 9, respectively, for the images ovule 1, 2, and 3 stacks, which is very different from the values used for our TS1 and TS2 sets.

### Evaluation metrics

#### Volume-averaged Jaccard index

The Jaccard index is a metric that estimates the similarity between 2 regions of labeled images, G and P, in terms of the intersection between them divided by their union [51]. Let us denote G_i_ and P_j_, these 2 overlapping regions, and their Jaccard index is defined as

$$JI(G_{i},\;P_{j})=\frac{\left|G_{i}\cap P_{j}\right|}{\left|G_{i}\cup P_{j}\right|},$$ (1)

where |X| denotes the volume of region X. We also define an asymmetric inclusion index metric between 2 regions defined as

$$I(G_{i},\;P_{j})=\frac{\left|G_{i}\cap P_{j}\right|}{\left|G_{i}\right|}$$ (2)

Given a set of *M* labeled regions {G_i_} in the ground truth image G, we associate with each cell region index i a cell region index *A*(*i*) = *j* in the image P containing the *N* predicted segmentations P_j_, using the Jaccard index

$$A(i)=arg\underset{j\in [2,N]}{max}JI(G_{i},\;P_{j})$$ (3)

Note that if the cell *G*_*i*_ has no intersection with any cell of P, we set *A*(*i*) = 0. Note that index i varies over [2,M] and j varies over [2,N] as background regions are not included in this estimation and in both the segmented and ground truth images (the background label is set as 1). Similarly, using the asymmetric index each region *G*_*i*_ of the image G is associated with one region index *B*(*i*) = *j* in the image P according to

$$B(i)=arg\underset{j\in [1,N]}{max}I(G_{i},\;P_{j})$$ (4)

Reciprocally, each region P_j_ of the image P is associated with one region index *B*′(*j*) = *i* in the image G:

$$B\prime (j)=arg\underset{i\in [1,M]}{max}I(P_{j},\;G_{i})$$ (5)

We then define an average metric between 2 images P and G, called volume-averaged Jaccard, which assesses how well the regions of 2 images overlap Index (VJI):

$$VJI(G,\;P)=\frac{\sum_{i=2}^{M}\left|G_{i}|JI(G_{i},P_{A(i)}\right)}{\sum_{i=2}^{M}\left|G_{i}\right|},$$ (6)

where G_i_ and P_j_ represent regions in, respectively, images G and P corresponding to either ground truth or predicted labeled cells. In this equation, it is assumed that *JI*(*G*_*i*_, *P*_0_) = 0 for any i. Background regions are not included in this estimation, and in both the segmented and ground truth images, the background label is set as 1 (index i starts at 2 in (6)).

**Rates of over- and undersegmentation to evaluate the quality of 3D segmentation methods.** The VJI is a measure that quantifies the degree of overlap between 2 segmentations, although it does not indicate whether the cell segmentation errors are due to over- or undersegmentation. In order to detect these different types of errors, we use the asymmetric index (2) to automatically determine a region-to-region correspondence map [52].

**Fig 21:**
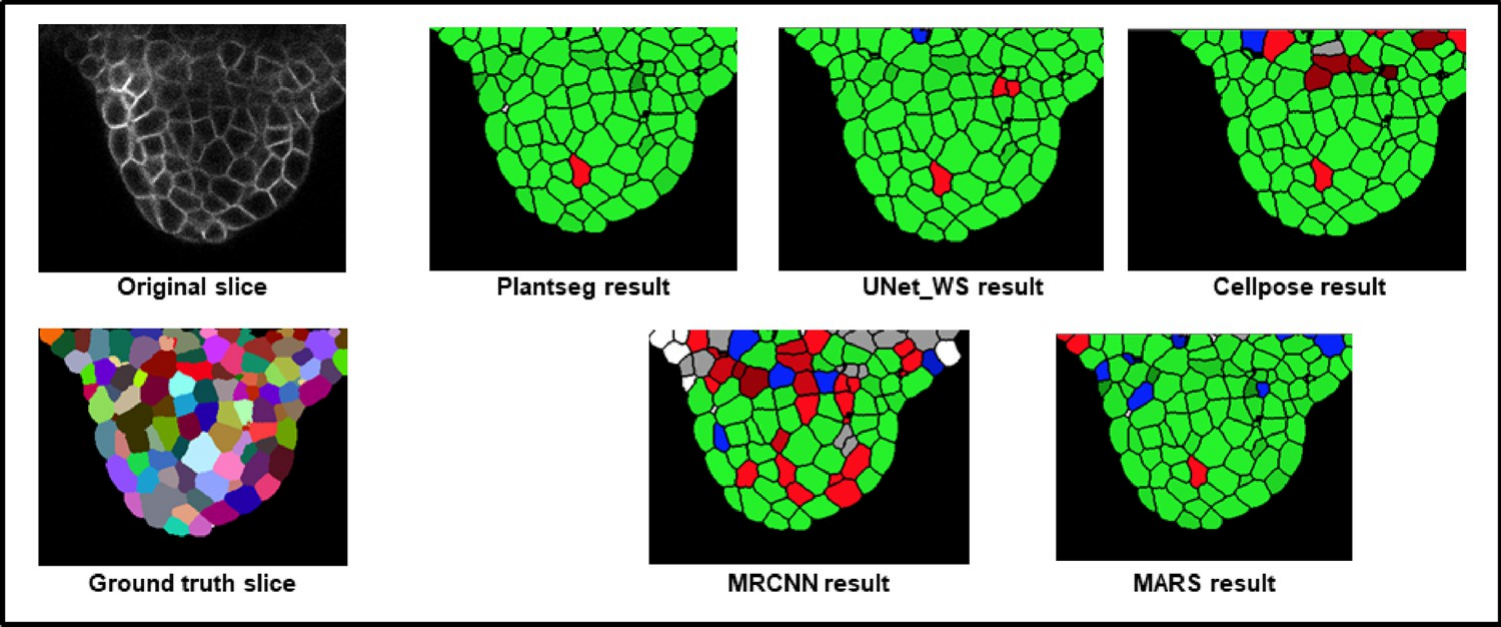
Segmentation quality metric [52] applied to outputs from 5 segmentation pipelines and types of errors displayed as a color map (on a common Z slice). The green cell regions represent regions of complete overlap between ground truth and predicted segmentations (i.e., regions of fully correct segmentation). Red regions represent over and blue regions represent undersegmentation errors. White regions are regions where cells were mistaken for background. The benefit of this metric is that it helps to estimate the rate of over- and undersegmentations as a volumetric statistics and as spatial distributions.

Using the previous inclusion index metrics, a correspondence map (Fig 21) is obtained by (1) making region-to-region association from the segmentation G to segmentation P then (2) repeating the procedure in the opposite direction, i.e., from image P to image G before (3) building the resulting reciprocal associations between sets of regions.

The 2 first steps consist of computing 2 values *B*(*i*) and *B*′(*j*) for each region index *i* of the first image and each region index *j* of the second image using (4) and (5), respectively. Using the previous computed indexes, set of pair of associated regions index between image P and G can be defined by

$$A=\{(i,j)|B(i)=j\;or\;B^{\prime }(j)=i)\}$$ (7)

Let us define the 2 subsets A(i)={j|(i,j)∈A}andA′(j)={i|(i,j)∈A} corresponding to the region indexes *j* associated with a given region index *i* and the region indexes *i* associated with a region index *j*, respectively. We then consider the different cases of resulting reciprocal mapping:

- one-to-one (exact match between *G*_*i*_ and *P*_*j*_) if A(i)={j}andA′(j)={i};
- one-to-many (oversegmentation of *G*_*i*_) *if* $|A(i)|>1\;and\;\forall_{j}\in A(i),\;A\prime (j)=\{i\}\;or\;A\prime (j)=\emptyset$;
- many-to-one (undersegmentation of *G*_*i*_) *if* $|A\prime (j)|>1\;and\;\forall_{i}\in A\prime (j),\;A(i)=\{j\}\;or\;A(i)=\emptyset$; and
- many-to-many otherwise.

It has to be noted that the correspondence involving the image background is treated separately, i.e., without considering a reciprocal association: The regions of segmentation G that maximize their inclusion with the background of the image P are associated and vice versa. From the resulting reciprocal mapping, a global rate of over- and undersegmentation can be calculated by counting the number of cells of the over- or undercorrespondence regions in the image P. Because of regions where tissues are not segmented in the ground truth segmentations, the predicted cells associated with the reference background are not counted in the final rate of over- and undersegmentation.

### Simulation of image artifacts

The effects of noise, blur, and intensity variations are simulated on the test set of 10 confocal images to evaluate their impact on the segmentation quality of the pipelines. The procedure for simulation of these artifacts are described below.

#### Image noise

The Gaussian noise was added to the images. The noise variable z is represented as the probability density function (PDF) P(z) and given by

$$P(z)=\frac{1}{\sigma \sqrt{2\pi }}e^{\frac{-{\left(z-\mu \right)}^{2}}{{2\sigma }^{2}}},$$ (8)

where μ is the mean, and σ is the standard deviation (or the square of the variance). Gaussian noise was generated with mean = 0.0 and 2 different values of noise variance ([0.04, 0.08]) and added to an image.

#### Image blur

To simulate motion blur, the test confocal image i(x,y) is convolved with a horizontal motion blur kernel w(dx, dy) of size 4 × 4 to get the final blurred image b(x,y).The convolution in the spatial domain is defined by Eq 9 below:

$$b(x,y)=w*i(x,y)=\sum_{dx=-a}^{a}\sum_{dy=-b}^{b}w(dx,dy)\;i(x+dx,y+dy)$$ (9)

**Fig 22:**
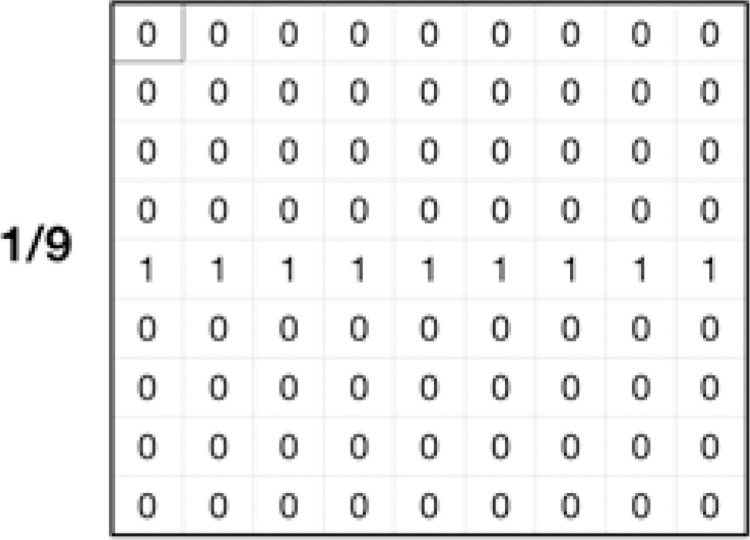
Kernel used for simulating the blur effect on confocal images.

The kernel used for simulating the image blur is a 9 × 9 horizontal motion blur kernel as shown in Fig 22 below.

#### Image intensity variations

**Fig 23:**
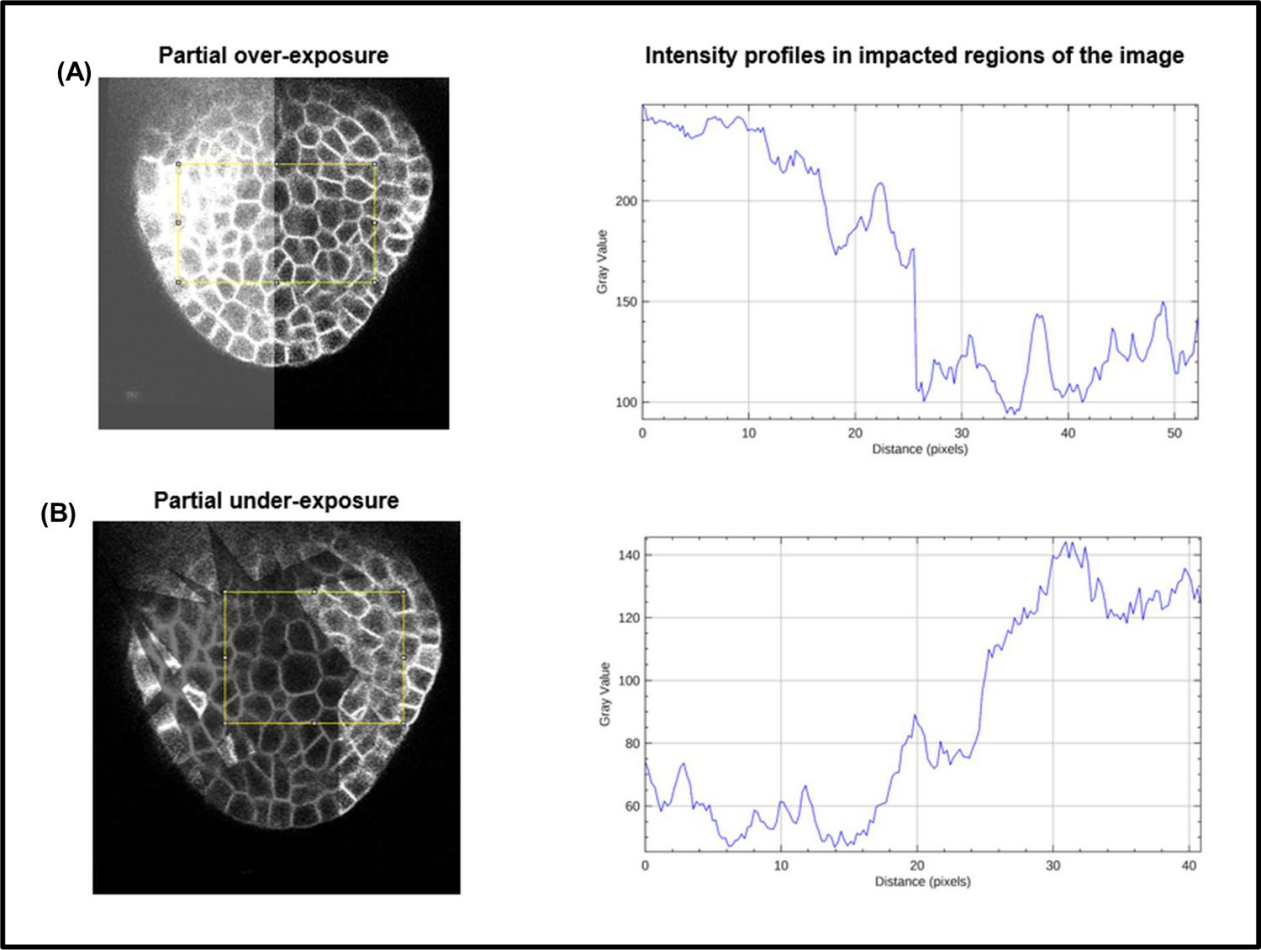
Modification of image intensity (inside selected area within the yellow box). **(A)** Image intensity transition under partial overexposure. **(B)** Image intensity variations due to imposition of underexposure.

Partially bright regions in microscopy images may be caused by inhomogeneous illumination sources and shadow effects are mostly caused by presence of light absorbing objects or obstructions [39,40,53,54]. To emulate the effect of intensity variations within an image, partial overexposure (Fig 23A) and random shadow regions (Fig 23B) are imposed (individually) on the test images. In order to impose the partial overexposure effect, for each Z slice of a given 3D test stack, a brightness mask is created, which is a 2D array having the same size as the x, y dimensions of the 3D stack. This 2D brightness mask array is filled with gray integer values of 255 for the left half of the mask array (Fig 23A). This brightness mask is then numerically added to each Z slice array with 30% transparency (using OpenCV function cv2.addweighted) to obtain the partially brightened image array as shown in Fig 23A.

For creating the randomly shadowed or underexposed regions, a shadow mask is created with random dark geometrical patch areas for each 2D Z slice of a 3D test stack. The dark patches are created by reducing image pixel intensities within the patch areas by a factor of 30% of the original intensity values. Fig 23A and 23B shows the intensity profiles within the yellow boundary boxes impacted due to the over- and underexposure effects, respectively. Within the overexposed regions, image intensities are higher than original values and vice versa for underexposed regions.

## Supporting information

S1 File Gitlab repository SegCompare. (DOCX)

S2 File Morphonet-based visualization of segmentation quality. (DOCX)

S3 File Data and model repositories. (DOCX)

S4 File Current research on DL-based instance segmentation techniques. DL, deep learning.
(DOCX)

S5 File Effect of retraining DL models with artifacts as data augmentation. DL, deep learning.
(DOCX)

S1 Fig Components of the SegCompare repository on Gitlab hosting the resources for training and evaluation of segmentation pipelines described in this paper. (TIFF)

S2 Fig Effect of retraining the residual 3D UNet model from Plantseg on datasets with augmentations (4 models in total: Original: model trained on unmodified images, Aug_under: model retrained with underexposed images, Aug_over: model retrained with overexposed images, Aug_mix: model retrained on dataset with over- and underexposed images). (TIF)

S3 Fig Results from retraining the 3D UNet model from UNet+WS pipeline on datasets with augmentations (4 models in total: Original: 3D UNet model trained on unmodified images, Aug_under: 3D UNet model retrained with underexposed images, Aug_over: 3D UNet model retrained with overexposed images, Aug_mix: 3D UNet model retrained on dataset containing both over- and underexposed images). (TIF)
