# Supplementary material for: Benchmarking of deep learning algorithms for 3D instance segmentation of confocal image datasets
Source: PLoS Comput Biol. 2022 Apr 14;18(4):e1009879. doi: 10.1371/journal.pcbi.1009879 (PMC9009699; doi:10.1371/journal.pcbi.1009879)
Supplement: S1 File — (DOCX) [file pcbi.1009879.s001.docx]

# **S1 File**

## **Gitlab repository SegCompare**

# SegCompare is an open repository of resources to train, test and evaluate multiple deep learning or non-deep learning algorithms (that are described in this article) and compare their segmentation quality both in a quantitative and visual manner. All the segmentation pipelines and evaluation methods in the repository are implemented using Python programming language. SegCompare resources are user friendly and contain:

# a) Steps for replicating deep learning and non-deep learning segmentation pipelines on custom user data. This includes steps for retraining the deep learning models used in this article as well as using them for directly segmenting user data.

# b) Methods for quantitative evaluation of segmentation quality for a given segmented image and its ground truth image

# c) Methods for 3D visualization of segmentation quality on the Morphological browser interface named Morphonet.

Link to Gitlab repository: <https://gitlab.inria.fr/mosaic/publications/seg_compare>

The Gitlab repository hosts a set of Jupyter notebooks for running the different tools presented in this work- such as implementation of the MARS segmentation pipeline, the segmentation evaluation metrics and also links to sample images with ground truth where the methods can be tested. Link to the notebooks are below:

<https://gitlab.inria.fr/mosaic/publications/seg_compare/-/tree/master/notebooks>

The repository also connects to a documentation page where details about each segmentation pipeline, segmentation evaluation and visualization methods are provided along with instructions for their installation. Further, links to all the datasets used in this work may be also found in this page.

Link to documentation page: <https://mosaic.gitlabpages.inria.fr/publications/seg_compare/>

**S1 Fig** Components of the SegCompare repository on Gitlab hosting the resources for training and evaluation of segmentation pipelines described in this paper.

The utilities of this repository (S1 Fig) are the following:

**For users wanting to segment their data:** They can directly use one of the trained models and pipelines described here to segment their data.

**For users designing a new 3D segmentation pipeline:** They can use the fully annotated image datasets and evaluation methods to estimate the quality of their method.

**For users having segmented data and expert ground truth:** They can use this repository to evaluate the quality of their segmentations with quantitative metrics and 3D visualizations.

The contents of SegCompare are as follows:

**Datasets:** A description of the confocal image datasets used for training and testing the segmentation pipelines described here. The links to the actual images are provided in S3.

**Installation instructions:** Each pipeline has its own library dependencies and therefore needs dedicated environments for running. For this, Python environments for each pipeline may be set up using yaml files, which are also provided in the SegCompare repository. The installation Instructions section provides detailed instructions to install environments for the segmentation pipelines, evaluation and visualization methods. After installing the environments, users can run the segmentation pipelines (for training or testing) or the evaluation and visualization methods.

**Segmentation pipelines:** Brief descriptions of each pipeline along with details of their pre- and post processing steps are presented here. Steps for dataset preparation for training the pipeline and links to training dataset are provided.

**Evaluation:** This section provides details of the segmentation evaluation metrics (Volume averaged Jaccard Index, Rates of over and under segmentation) and Jupyter (Python) notebooks for implementing them on a pair of segmented images. For evaluating segmentations a user must have a segmented image and corresponding ground truth segmentation (currently .tif format is supported for images). Sample segmented and ground truth data are in the data repository described in S3 File.

**3D Visualization:** This section describes how the browser based Morphological data visualization platform Morphonet may be used for visualizing segmentation quality. A Jupyter notebook is provided which contains the full implementation of the visualization pipeline starting from a segmented image. For this visualization, results from the Jaccard Index evaluation notebook are required and the full workflow is documented in the repository. Links to sample meshes and datasets for uploading to Morphonet are provided along with demo videos (described in S3 File).

**Downloads:** This section contains links to Jupyter notebooks for implementations of the MARS pipeline, segmentation evaluation metrics and 3D visualizations. Sample CSV files with Jaccard index estimates and corresponding mesh files for uploading to Morphonet are in the data repository (see S3 File) .

Link to Jupyter Notebooks on Gitlab:

<https://gitlab.inria.fr/mosaic/publications/seg_compare/-/tree/master/notebooks>
