# Supplementary material for: Benchmarking of deep learning algorithms for 3D instance segmentation of confocal image datasets
Source: PLoS Comput Biol. 2022 Apr 14;18(4):e1009879. doi: 10.1371/journal.pcbi.1009879 (PMC9009699; doi:10.1371/journal.pcbi.1009879)
Supplement: S2 File — (DOCX) [file pcbi.1009879.s002.docx]

# **S2 File**

## **Morphonet based visualization of segmentation quality**

To visualize quality of the five segmentation pipelines on Morphonet, users can click the link below which will directly take them to an uploaded dataset with segmentation quality information from the 5 pipelines (MorphoNet works best with Chrome or Firefox) :

<https://morphonet.org/icRos2mO>

At first only a 3D mesh of the image is displayed. Next, to visualize Jaccard Index info for the pipelines, click on Info-> Click on Info name-> Set colormap. This superposes the VJI values as color-mapped information on the mesh. Multiple meshes and multiple information may be uploaded in this manner.

To upload and visualize information on Morphonet, users need to create an account on Morphonet.org by clicking the “Signup” option on the page or use the guest account provided with this paper. To use this guest account, users may login using : **username:** guest, **password:** guest2021.

To use the Morphonet platform for uploading new data, after logging in to Morphonet, the users may upload meshes for multiple time points and corresponding information to superpose on the meshes. For visualizing segmentation quality on a cell by cell basis, users need to first compute the Volume -averaged Jaccard Index metric using the Volume averaged Jaccard index evaluation.ipynb (may be found under Downloads section of the SegCompare repository), using as input a segmented image and corresponding ground truth segmentation. The output of the Jupyter notebook is a CSV file containing Volume averaged Jaccard Index measure for each cell. This CSV file along with the ground truth segmented image may be used with the 3D_visualization.ipynb notebook (also under Downloads/Notebooks in the SegCompare repository ) to do a one step uploading of mesh and numerical information on Morphonet. After running this notebook, users can go to the Morphonet.org page, navigate to their dataset to visualize it. Sample videos demonstrating these operations may be found in our figshare repository as described below under “Videos”.
