# Supplementary material for: Benchmarking of deep learning algorithms for 3D instance segmentation of confocal image datasets
Source: PLoS Comput Biol. 2022 Apr 14;18(4):e1009879. doi: 10.1371/journal.pcbi.1009879 (PMC9009699; doi:10.1371/journal.pcbi.1009879)
Supplement: S3 File — (DOCX) [file pcbi.1009879.s003.docx]

# **S3 File**

# **Data and model repositories**

# The training and test datasets used in this work are available online. The training dataset of shoot apical meristems may be found under: <https://www.repository.cam.ac.uk/handle/1810/262530>

The full set of test images of floral meristems may be found at:

<https://www.repository.cam.ac.uk/handle/1810/318119>

We have used selected images from the above repository. Within this repository, there are overall 6 meristems named FM1-FM6. From this, we selected two meristems FM1 (18 time points) and FM6(5 time points). Out of these, we chose images corresponding to 6 time points from FM1 and 4 from FM6 to create our test dataset. In our test dataset, we renamed the images from FM1 as TS1-00h, TS1-24h, TS1-32h, TS1-72h, TS1-120h, TS1-132h according to the value of the timepoints of the images. The term TS1 represents “Test set 1” . Similarly the images from FM6 are named as TS2-26h, TS2-44h, TS2-56h, TS2-69h where TS2 represents “Test set 2” and the numbers correspond to the timepoints.

A repository of materials generated as part of this study may be found at

LINK: <https://figshare.com/projects/3D_segmentation_and_evaluation/101120>

This repository (3D Segmentation and evaluation) contains the trained models for each of the pipelines, meshes for uploading to Morphonet and corresponding segmentation accuracy files in CSV format. The test images used in this work (TS1-00h, TS1-24h, TS1-32h, TS1-72h, TS1-120h, TS1-132h, TS2-26h, TS2-44h, TS2-56h, TS2-69h ) may be found as .tif images under “Test dataset” within this repository.

(Link <https://figshare.com/articles/dataset/Test_dataset/16602323>)

The overall contents of the repository are the following:

**Trained deep learning models:** The models trained in the four deep learning pipelines are provided. Instructions for running them are in the Gitlab repository (S1 File).

**Original stacks and segmented data:** Segmented confocal stacks by each of the five pipelines are provided along with ground truth stacks for each. Users may test the segmentation evaluation methods using them. Details of using the evaluation function are in the Gitlab (S1 File).

**Meshes for Morphonet:** Example meshes that might be uploaded to Morphonet are included. Users may test the Morphonet visualization using these and the cellwise VJI values (saved in CSV files). Procedure for the visualization is provided in the Gitlab repository.

**Accuracy results:** Cellwise VJI values saved in CSV files are provided for each pipeline. These may be used for projection on Morphonet for 3D visualization of segmentation quality.

**Videos:** Videos (.mp4 format) showing examples on how to use the Morphonet based 3D visualization method on a sample test image, videos showing sample training and test data.

**Test dataset:** The test images used in this work (TS1-00h, TS1-24h, TS1-32h, TS1-72h, TS1-120h, TS1-132h, TS2-26h, TS2-44h, TS2-56h, TS2-69h )
