# Supplementary material for: Benchmarking of deep learning algorithms for 3D instance segmentation of confocal image datasets
Source: PLoS Comput Biol. 2022 Apr 14;18(4):e1009879. doi: 10.1371/journal.pcbi.1009879 (PMC9009699; doi:10.1371/journal.pcbi.1009879)
Supplement: S4 File — DL, deep learning. (DOCX) [file pcbi.1009879.s004.docx]

# **S4 File**

## **Current research on deep learning based instance segmentation techniques**

In complement to the survey given in the introduction, we provide here a more extensive overview of the existing deep learning based segmentation methods identifying the major trends of research in this rapidly evolving field. The focus of the survey is on methods that are developed for instance segmentation of images and papers for non-image datasets are excluded. We identified different categories of pipelines, which have been developed to address specific challenges. The research works belonging to each category are discussed below. The main purpose is to illustrate the existing diversity, rather than giving all the details of the individual methods, which is out of the scope of this article.

**Pipelines for end to end 3D instance segmentation.** As discussed in the introduction of the main text, end to end 3D (3D input, 3D output) segmentation pipelines have been implemented using either UNet, residual UNet or MaskRCNN architectures. For more details on the UNet and Residual UNet architectures see [[1]](https://sciwheel.com/work/citation?ids=6152448&pre=&suf=&sa=0) [[2]](https://sciwheel.com/work/citation?ids=10387774&pre=&suf=&sa=0) and [[3]](https://sciwheel.com/work/citation?ids=4942671&pre=&suf=&sa=0) for MaskRCNN. Besides the pipelines used here (Plantseg [[4]](https://sciwheel.com/work/citation?ids=9355538&pre=&suf=&sa=0), UNet+WS [[5]](https://sciwheel.com/work/citation?ids=9720077&pre=&suf=&sa=0) and Cellpose [[6]](https://sciwheel.com/work/citation?ids=10164215&pre=&suf=&sa=0)), ([[7]](https://sciwheel.com/work/citation?ids=8507977&pre=&suf=&sa=0)) proposed a method which uses 3D images of A. Thaliana and time lapse images of leaf epidermal tissue for training a 3D UNet. This UNet extracts cell boundaries that are processed using 3D watershed along with conditional random fields (a prediction concept which uses contextual information from previous labels).

**Deep learning algorithms for 2D instance segmentation:** Mask RCNN is widely used for highly accurate 2D instance segmentation. [[8]](https://sciwheel.com/work/citation?ids=10386437&pre=&suf=&sa=0) tested two deep learning architectures for 2D nucleus segmentation i.e. a feature pyramid network (FPN) and a Mask RCNN. This study indicates that Mask RCNN gave superior results. [[9]](https://sciwheel.com/work/citation?ids=10387775&pre=&suf=&sa=0) used a modification of the basic MRCNN to perform multi-organ segmentation of human esophageal cancer CT images and mitigate effects of fuzzy organ boundaries and diverse organ shapes in the images. The additional features in the proposed algorithm include a pre-background classification step to improve boundary predictions and use of a custom loss function. In [[10]](https://sciwheel.com/work/citation?ids=10387762&pre=&suf=&sa=0) a modified Mask RCNN architecture termed as Panoptic Domain Adaptive Mask R-CNN is developed to achieve unsupervised segmentation of nuclei from histopathology images.

UNets are also used for 2D image segmentation. In [[11]](https://sciwheel.com/work/citation?ids=6535414&pre=&suf=&sa=0) a UNet based module followed by post processing steps of thresholding and watershed is used to predict locations of the cells and their nuclei. Multiple deep learning architectures based on UNet, modified UNet and Mask RCNN are tested for 2D nuclear image segmentation in [[12]](https://sciwheel.com/work/citation?ids=10386463&pre=&suf=&sa=0) and the Mask RCNN architecture was found to outperform the UNet based models in the 2D segmentation task. It may be noted that for evaluation [[8]](https://sciwheel.com/work/citation?ids=10386437&pre=&suf=&sa=0) use F1-score, while [[12]](https://sciwheel.com/work/citation?ids=10386463&pre=&suf=&sa=0) use under/oversegmentation and aggregated Jaccard index.

**Deep learning based segmentation pipelines for specific purposes in bioimaging**

**a. Deep learning for cell segmentation and tracking:** Deep learning pipelines for instance segmentation coupled with cell tracking have been proposed in several works, such as [[13]](https://sciwheel.com/work/citation?ids=10389336&pre=&suf=&sa=0) where the pipeline makes predictions for every cell instance in videos, as well produces temporally connected instance segmentations. Cell instance segmentation in calcium imaging videos is described in [[14]](https://sciwheel.com/work/citation?ids=10387747&pre=&suf=&sa=0) which uses temporal information to estimate pixel-wise correlation and shape information to identify cells and classify active and non-active cells. [[15]](https://sciwheel.com/work/citation?ids=10387748&pre=&suf=&sa=0) also proposes a modified UNet based model which can be used for tracking cells while dealing with challenging conditions such as crowded cell regions, poor image quality and on data with missing annotations. The method is tested on cell images from mouse muscle stem cells, HeLa cells, and images from developing embryos. Other approaches for implementation of cell segmentation and tracking include [[16]](https://sciwheel.com/work/citation?ids=10387755&pre=&suf=&sa=0) and [[17]](https://sciwheel.com/work/citation?ids=9056097&pre=&suf=&sa=0). The latter uses two UNet models to create an architecture named DELTA to first segment the cells followed by tracking lineage reconstruction from time lapse videos of E. coli cells in fluidic medium.

**b. Pipelines for addressing sparse annotations and small training datasets:** For training of deep learning based segmentation models, annotated ground truth data is essential. However, expert annotation of biomedical images (especially 3D datasets) is a highly labor intensive and time consuming process. For this reason, several deep learning pipelines have been developed which can work with sparsely annotated data. These include the method described by [[18]](https://sciwheel.com/work/citation?ids=8501888&pre=&suf=&sa=0) which uses only a few fully annotated voxel instances to segment a full 3D stack. In [[19]](https://sciwheel.com/work/citation?ids=10387765&pre=&suf=&sa=0) it is demonstrated how varying the contrasts of cell boundaries and a new loss function (weighted cross entropy) could be useful to obtain high accuracy segmentations when a 3D UNet model trained with a small and sparsely annotated training dataset. The issue of sparse annotations is also addressed in works like [[20]](https://sciwheel.com/work/citation?ids=10387749&pre=&suf=&sa=0) and [[21]](https://sciwheel.com/work/citation?ids=6747447&pre=&suf=&sa=0)**.**

**c. Pipelines for segmenting images with densely packed cells/tissues :** Cell instance segmentation in images where cells appear in dense clusters or in overlapping manner is a common research problem. It is quite challenging as there are high chances of errors in separating each cell. Specially designed deep learning models for segmenting densely packed cell regions are reported in works like [[22]](https://sciwheel.com/work/citation?ids=10386459&pre=&suf=&sa=0). It uses an object detection module called a feature pyramid network, which apparently outperforms MRCNN in this task. The feature pyramid network extracts information of the same image at different scales, in this case the cells and the subcellular nuclear scale. In [[23]](https://sciwheel.com/work/citation?ids=10387744&pre=&suf=&sa=0) a hybrid architecture combining UNet and MRCNN is proposed to address effects of crowded and variable sized objects, named Nuclei Segmentation Toolset or NuSeT for nuclei segmentation. The U-Net here is used for semantic level segmentation, the modified MRCNN predicts the instance bounding boxes based on the UNet outputs which are then finally used as seeds for watershed segmentation. [[24]](https://sciwheel.com/work/citation?ids=10387766&pre=&suf=&sa=0) uses a combination of two CNNs, that provide a semantic segmentation of nuclear material, followed by a final instance level segmentation of the individual nuclei. The research in [[25]](https://sciwheel.com/work/citation?ids=10387757&pre=&suf=&sa=0) performs 3 class classifications on a human tissue image dataset to distinguish between cell boundaries, inside and outside of dense nuclei regions. A new CNN architecture named HoVer-Net is presented in [[26]](https://sciwheel.com/work/citation?ids=10387768&pre=&suf=&sa=0) for instance level segmentation of nuclei from histological images where they appear overlapped with each other. This method can further classify the type of nuclei, e.g.the type of cells-such as between tumor and lymphocyte cells from which the nuclei are obtained. Another method specially designed for dense cell clusters in images is [[27]](https://sciwheel.com/work/citation?ids=10386434&pre=&suf=&sa=0) which uses two UNet based deep models to predict pixels belonging to the cell regions. Using this output, a final watershed based segmentation is implemented.

**d. Pipelines for segmenting special cell shapes:** In many biological datasets, cells or tissues could have morphologically complex, e.g. very thin or elongated shapes that are difficult to segment by generic pipelines built for regular spherical cell shapes. Deep learning pipelines, with specific DL architectures for segmenting this kind of data have been developed. This includes the method described by [[28]](https://sciwheel.com/work/citation?ids=10386460&pre=&suf=&sa=0) which can perform precise segmentation of neural cells which have unconventional structures while also countering challenges like cell division and unclear cell boundaries. An approach combining object detection and segmentation is described in [[29]](https://sciwheel.com/work/citation?ids=10386461&pre=&suf=&sa=0) which is successful in high precision detection of small scale and narrow structures of neural cells. Other works include [[30]](https://sciwheel.com/work/citation?ids=7954425&pre=&suf=&sa=0) for the segmentation of glial cells, a deep learning model named DeepEM3D-Net in [[31]](https://sciwheel.com/work/citation?ids=9909726&pre=&suf=&sa=0) for segmenting 3D neurite images and [[32]](https://sciwheel.com/work/citation?ids=10387773&pre=&suf=&sa=0) for segmentation of diversely shaped human organs in images.

**Conclusions.**

From this literature survey, the following aspects are observed 1) UNet and Mask RCNN are two most common deep learning architectures that are currently used for instance segmentation of biological images 2) The number of works on end to end 3D deep learning for instance segmentation is much lower than that for 2D image datasets. 3) the existing segmentation pipelines have been trained on a wide variety of datasets (plant and animal tissue, cell and nuclei; 2D and 3D still images, videos, time-lapse images) and therefore it is not possible to determine their relative performance levels. 4) For many of the methods surveyed it is not possible to reproduce the pipeline as they are not open source or do not allow retraining. 5) There exists a shortage of large 3D annotated image datasets on plant and animal tissues which are publicly available.

**References**

[1. Falk T, Mai D, Bensch R, Çiçek Ö, Abdulkadir A, Marrakchi Y, et al. U-Net: deep learning for cell counting, detection, and morphometry. Nat Methods. 2019;16: 67–70. doi:10.1038/s41592-018-0261-2](https://sciwheel.com/work/bibliography/6152448)

[2. Zhu N, Liu C, Singer ZS, Danino T, Laine A, Guo J. Segmentation with Residual Attention U-Net and an Edge-Enhancement Approach Preserves Cell Shape Features. ArXiv. 2020;abs/2001.05548.](https://sciwheel.com/work/bibliography/10387774)

[3. He K, Gkioxari G, Dollar P, Girshick R. Mask R-CNN. IEEE International Conference on Computer Vision (ICCV). IEEE; 2017. pp. 2980–2988. doi:10.1109/ICCV.2017.322](https://sciwheel.com/work/bibliography/4942671)

[4. Wolny A, Cerrone L, Vijayan A, Tofanelli R, Barro AV, Louveaux M, et al. Accurate and versatile 3D segmentation of plant tissues at cellular resolution. eLife. 2020;9. doi:10.7554/eLife.57613](https://sciwheel.com/work/bibliography/9355538)

[5. Eschweiler D, Spina TV, Choudhury RC, Meyerowitz E, Cunha A, Stegmaier J. CNN-based preprocessing to optimize watershed-based cell segmentation in 3D confocal microscopy images. 2019 IEEE 16th International Symposium on Biomedical Imaging (ISBI 2019). IEEE; 2019. pp. 223–227. doi:10.1109/ISBI.2019.8759242](https://sciwheel.com/work/bibliography/9720077)

[6. Stringer C, Wang T, Michaelos M, Pachitariu M. Cellpose: a generalist algorithm for cellular segmentation. Nat Methods. 2021;18: 100–106. doi:10.1038/s41592-020-01018-x](https://sciwheel.com/work/bibliography/10164215)

[7. Jiang J, Kao P-Y, Belteton SA, Szymanski DB, Manjunath BS. Accurate 3D cell segmentation using deep features and CRF refinement. 2019 IEEE International Conference on Image Processing (ICIP). IEEE; 2019. pp. 1555–1559. doi:10.1109/ICIP.2019.8803095](https://sciwheel.com/work/bibliography/8507977)

[8. Zaki G, Gudla PR, Lee K, Kim J, Ozbun L, Shachar S, et al. A deep learning pipeline for nucleus segmentation. BioRxiv. 2020. doi:10.1101/2020.04.14.041020](https://sciwheel.com/work/bibliography/10386437)

[9. Shu J-H, Nian F-D, Yu M-H, Li X. An Improved Mask R-CNN Model for Multiorgan Segmentation. Mathematical Problems in Engineering. 2020;2020: 1–11. doi:10.1155/2020/8351725](https://sciwheel.com/work/bibliography/10387775)

[10. Liu D, Zhang D, Song Y, Zhang F, O’Donnell L, Huang H, et al. Unsupervised Instance Segmentation in Microscopy Images via Panoptic Domain Adaptation and Task Re-Weighting. 2020 IEEE/CVF Conference on Computer Vision and Pattern Recognition (CVPR). 2020; 4242–4251.](https://sciwheel.com/work/bibliography/10387762)

[11. Al-Kofahi Y, Zaltsman A, Graves R, Marshall W, Rusu M. A deep learning-based algorithm for 2-D cell segmentation in microscopy images. BMC Bioinformatics. 2018;19: 365. doi:10.1186/s12859-018-2375-z](https://sciwheel.com/work/bibliography/6535414)

[12. Kromp F, Fischer L, Bozsaky E, Ambros I, Doerr W, Taschner-Mandl S, et al. Deep Learning architectures for generalized immunofluorescence based nuclear image segmentation. ArXiv. 2019;abs/1907.12975.](https://sciwheel.com/work/bibliography/10386463)

[13. Payer C, Štern D, Feiner M, Bischof H, Urschler M. Segmenting and tracking cell instances with cosine embeddings and recurrent hourglass networks. Med Image Anal. 2019;57: 106–119. doi:10.1016/j.media.2019.06.015](https://sciwheel.com/work/bibliography/10389336)

[14. Kirschbaum E, Bailoni A, Hamprecht FA. Disco: deep learning, instance segmentation, and correlations for cell segmentation in calcium imaging. In: Martel AL, Abolmaesumi P, Stoyanov D, Mateus D, Zuluaga MA, Zhou SK, et al., editors. Medical image computing and computer assisted intervention – MICCAI 2020: 23rd international conference, lima, peru, october 4–8, 2020, proceedings, part V. Cham: Springer International Publishing; 2020. pp. 151–162. doi:10.1007/978-3-030-59722-1_15](https://sciwheel.com/work/bibliography/10387747)

[15. Scherr T, Löffler K, Böhland M, Mikut R. Cell segmentation and tracking using CNN-based distance predictions and a graph-based matching strategy. PLoS ONE. 2020;15: e0243219. doi:10.1371/journal.pone.0243219](https://sciwheel.com/work/bibliography/10387748)

[16. Wang C, Zhang X, Choi HJ, Lin B, Yu Y, Whittle C, et al. Deep learning pipeline for cell edge segmentation of time-lapse live cell images. bioRxiv. 2019.](https://sciwheel.com/work/bibliography/10387755)

[17. Lugagne J-B, Lin H, Dunlop MJ. DeLTA: Automated cell segmentation, tracking, and lineage reconstruction using deep learning. PLoS Comput Biol. 2020;16: e1007673. doi:10.1371/journal.pcbi.1007673](https://sciwheel.com/work/bibliography/9056097)

[18. Zhao Z, Yang L, Zheng H, Guldner IH, Zhang S, Chen DZ. Deep learning based instance segmentation in 3D biomedical images using weak annotation. In: Frangi AF, Schnabel JA, Davatzikos C, Alberola-López C, Fichtinger G, editors. Medical Image Computing and Computer Assisted Intervention – MICCAI 2018: 21st International Conference, Granada, Spain, September 16-20, 2018, Proceedings, Part IV. Cham: Springer International Publishing; 2018. pp. 352–360. doi:10.1007/978-3-030-00937-3_41](https://sciwheel.com/work/bibliography/8501888)

[19. Guerrero-Peña FA, Marrero-Fernández PD, Tsang IR, Cunha A. A Weakly Supervised Method for Instance Segmentation of Biological Cells. ArXiv. 2019;abs/1908.09891.](https://sciwheel.com/work/bibliography/10387765)

[20. Dawoud Y, Hornauer J, Carneiro G, Belagiannis V. Few-Shot Microscopy Image Cell Segmentation. ArXiv. 2020;abs/2007.01671.](https://sciwheel.com/work/bibliography/10387749)

[21. Arbelle A, Raviv TR. Microscopy cell segmentation via adversarial neural networks. 2018 IEEE 15th International Symposium on Biomedical Imaging (ISBI 2018). IEEE; 2018. pp. 645–648. doi:10.1109/ISBI.2018.8363657](https://sciwheel.com/work/bibliography/6747447)

[22. Korfhage N, Mühling M, Ringshandl S, Becker A, Schmeck B, Freisleben B. Detection and segmentation of morphologically complex eukaryotic cells in fluorescence microscopy images via feature pyramid fusion. PLoS Comput Biol. 2020;16: e1008179. doi:10.1371/journal.pcbi.1008179](https://sciwheel.com/work/bibliography/10386459)

[23. Yang L, Ghosh RP, Franklin JM, Chen S, You C, Narayan RR, et al. NuSeT: A deep learning tool for reliably separating and analyzing crowded cells. PLoS Comput Biol. 2020;16: e1008193. doi:10.1371/journal.pcbi.1008193](https://sciwheel.com/work/bibliography/10387744)

[24. Vu QD, Graham S, Kurc T, To MNN, Shaban M, Qaiser T, et al. Methods for segmentation and classification of digital microscopy tissue images. Front Bioeng Biotechnol. 2019;7: 53. doi:10.3389/fbioe.2019.00053](https://sciwheel.com/work/bibliography/10387766)

[25. Kumar N, Verma R, Anand D, Zhou Y, Onder OF, Tsougenis E, et al. A Multi-Organ Nucleus Segmentation Challenge. IEEE Trans Med Imaging. 2020;39: 1380–1391. doi:10.1109/TMI.2019.2947628](https://sciwheel.com/work/bibliography/10387757)

[26. Graham S, Vu QD, Raza SEA, Azam A, Tsang YW, Kwak JT, et al. Hover-Net: Simultaneous segmentation and classification of nuclei in multi-tissue histology images. Med Image Anal. 2019;58: 101563. doi:10.1016/j.media.2019.101563](https://sciwheel.com/work/bibliography/10387768)

[27. Lux F, Matula P. Cell Segmentation by Combining Marker-Controlled Watershed and Deep Learning. ArXiv. 2020;abs/2004.01607.](https://sciwheel.com/work/bibliography/10386434)

[28. Yi J, Wu P, Jiang M, Huang Q, Hoeppner DJ, Metaxas DN. Attentive neural cell instance segmentation. Med Image Anal. 2019;55: 228–240. doi:10.1016/j.media.2019.05.004](https://sciwheel.com/work/bibliography/10386460)

[29. Yi J, Wu P, Hoeppner DJ, Metaxas D. Pixel-wise neural cell instance segmentation. 2018 IEEE 15th International Symposium on Biomedical Imaging (ISBI 2018). IEEE; 2018. pp. 373–377. doi:10.1109/ISBI.2018.8363596](https://sciwheel.com/work/bibliography/10386461)

[30. Yang L, Zhang Y, Guldner IH, Zhang S, Chen DZ. 3D Segmentation of Glial Cells Using Fully Convolutional Networks and k-Terminal Cut. In: Ourselin S, Joskowicz L, Sabuncu MR, Unal G, Wells W, editors. Medical Image Computing and Computer-Assisted Intervention – MICCAI 2016. Cham: Springer International Publishing; 2016. pp. 658–666. doi:10.1007/978-3-319-46723-8_76](https://sciwheel.com/work/bibliography/7954425)

[31. Zeng T, Wu B, Ji S. DeepEM3D: approaching human-level performance on 3D anisotropic EM image segmentation. Bioinformatics. 2017;33: 2555–2562. doi:10.1093/bioinformatics/btx188](https://sciwheel.com/work/bibliography/9909726)

[32. Gu Z, Cheng J, Fu H, Zhou K, Hao H, Zhao Y, et al. CE-Net: Context Encoder Network for 2D Medical Image Segmentation. IEEE Trans Med Imaging. 2019;38: 2281–2292. doi:10.1109/TMI.2019.2903562](https://sciwheel.com/work/bibliography/10387773)
