# Supplementary material for: Benchmarking of deep learning algorithms for 3D instance segmentation of confocal image datasets
Source: PLoS Comput Biol. 2022 Apr 14;18(4):e1009879. doi: 10.1371/journal.pcbi.1009879 (PMC9009699; doi:10.1371/journal.pcbi.1009879)
Supplement: S5 File — DL, deep learning. (DOCX) [file pcbi.1009879.s005.docx]

# **S5 File**

**Effect of retraining deep learning models with artifacts as data augmentation**

It is observed from the results in section “***Strategy 3: Evaluating pipelines on synthetically modified images***” that the accuracies of deep learning based segmentation models deteriorate when they are subjected to images with previously unseen artifacts such as over and under-exposure, noise and blur. Out of these, the effects of partial under and overexposure are seen to significantly impact the Plantseg and UNet watershed pipelines. This may be observed from the plots of Fig 7-9 in the main text.

It may however be noted that the deep learning models respective to these two pipelines (i.e. 3D Residual UNet of Plantseg and 3D UNet model of UNet+Watershed) were initially trained on image datasets that did not contain the partial over or under exposure artifacts in them. Therefore the question arises as to what happens when the training set is augmented with images that contain the overexposure and/or the underexposure artifact? For studying this, a new set of experiments were performed by retraining the two deep learning models of these pipelines. In the new experiments the models were retrained by augmenting the training dataset with images where the under and overexposure effects are introduced. Subsequently, the effect of inclusion of artifacts (as augmentations) in training data are investigated. The details of these experiments for each pipeline (Plantseg, UNet+Watershed) are provided below along with the results.

***Experiment 1: Retraining 3D residual UNet of Plantseg by augmenting with over-exposure***

In this experiment, the training dataset for the 3D Residual UNet model of Plantseg was augmented with images where the partial overexposure effect is introduced. This augmented training set had normal training stacks as well as stacks with overexposure effects. The function to create the over-exposure was the same as described in this paper under section “Simulation of image artifacts” under “Image intensity variations”.

The results from this experiment are shown in S2 Fig below. The plot shows the comparison between results obtained with the original model alongside those from the retrained model. The retrained model (Aug_Over) was used to segment 3 types of images which include- normal images, images with over-exposure and images with under-exposure. It is observed that as a result of training the model with this augmentation, the segmentation accuracy of the model increases significantly when it is used to segment images with over-exposure as well as normal images. However, the accuracy of segmenting images with under-exposure using this newly trained model is lower than that of the original model. Thus It may be concluded that this model learns to segment both normal and over-exposed data with high accuracy but does not produce good results on under-exposed images.

***Experiment 2: Retraining 3D residual UNet of Plantseg by augmenting with under-exposure***

In this experiment, the training dataset for the 3D Residual UNet model was augmented with images where the partial under-exposure effect is introduced. This augmented training set had both normal training stacks and stacks with under-exposure effect. The function to create the under-exposure was the same as described in Section “Simulation of image artifacts” above. The retrained model (Aug_Under) was then used to segment test data to evaluate its performance. For testing, the same test dataset was used- that is normal 10 test stacks as shown in Fig 4A (main text) and the same 10 stacks simulated with over-exposure and under-exposure (i.e 30 test stacks).

The results from this experiment are shown in S2 Fig below. The plot shows the comparison between results obtained with the original model alongside those from the retrained model. As a result of this training data augmentation, the segmentation accuracy of the model increases for under-exposed test data and it also segments normal images with as high accuracy as the original model. The improvement in accuracy for over-exposed images is not as high as the model obtained in Experiment 1. It may be concluded that this model learns to segment both normal and under-exposed data with high accuracy and also produces good results on over-exposed images.

***Experiment 3: Retraining 3D residual UNet of Plantseg pipeline by augmenting with both over and under-exposure***

In this experiment, the training dataset for the 3D Residual UNet model was augmented with both over and under-exposed images. This augmented training set therefore had normal training stacks, stacks with over-exposure and stacks with under exposure effect. As before, the functions to create the over and under-exposure were the same as described in Section “Simulation of image artifacts” above. For testing, also three types of images were used- that is normal 10 test stacks as shown in Fig 4A of main text and the same 10 stacks simulated with over-exposure and under-exposure (i.e total of 30 stacks).

The results from this experiment are shown in S2 Fig. The plot shows the comparison between results obtained with the original model with those from the retrained model. This model produces high accuracy for all three types of images-i.e normal, over and underexposed ones. The mixed augmentation thus provides an overall improvement in the segmentation quality of the residual 3D UNet model of the Plantseg pipeline.

**S2 Fig** Effect of retraining the residual 3D UNet model from Plantseg on datasets with augmentations. (4 models in total: Original: model trained on unmodified images, Aug_under: model retrained with under-exposed images, Aug_over: model retrained with overexposed images, Aug_mix: model retrained on dataset with over and under-exposed images).

***Experiment 4: Retraining 3D UNet of UNet +WS pipeline by augmenting with overexposure***

In this experiment, the training dataset for the 3D UNet model of the UNet +WS was augmented with images where the partial overexposure effect is introduced in the same way as done in Experiment 1 for Plantseg described above. The results from this experiment are shown in S3 Fig below. The plot shows the comparison between results obtained with the original model alongside those from the retrained model. The retrained model (Aug_Over) was used to segment 3 types of images which include- normal images, images with over-exposure and images with under-exposure. It is observed that as a result of training the model with this augmentation, the segmentation accuracy of the model only increases when it is used to segment images with over-exposure. However, the accuracy level of this model for segmenting normal images is much lower compared to the original model. Similarly, accuracy of the retrained model is lower than the original model when trying to segment under exposed images.

Thus it is observed that with over exposure augmentation, the retrained model learns to better segment overexposed images but fails to generalize while segmenting both normal and under-exposed images. This is different from what is observed with the Plantseg pipeline above.

***Experiment 5: Retraining 3D UNet of UNet +WS pipeline by augmenting with underexposure***

In this experiment, the training dataset for the 3D UNet model was augmented with images influenced with the partial under-exposure effect. This augmented training set had both normal training stacks and stacks with under-exposure (same as done in Experiment 2 above). The retrained model (Aug_Under) was then used to segment test data to evaluate its performance. For testing, the same test dataset of 30 stacks was used as done in the experiments above- that is 10 normal test stacks, same 10 stacks simulated with over-exposure and under-exposure.

**S3 Fig** Results from retraining the 3D UNet model from UNet+WS pipeline on datasets with augmentations (4 models in total: Original: 3D UNet model trained on unmodified images, Aug_under: 3D UNet model retrained with under-exposed images, Aug_over: 3D UNet model retrained with overexposed images, Aug_mix: 3D UNet model retrained on dataset containing both over and under-exposed images).

The results from this experiment are in S3 Fig which shows the comparison between results obtained with the original model beside those from the retrained model on augmented data. It is seen that as a result of augmenting training data with underexposed images, the segmentation accuracy of the model increases for all three types of test images. Thus the retrained model performs better than the original model as it provides higher accuracy on normal images as well as on images with under and overexposure. Thus it may be stated that augmenting with under-exposure improves the generalizability of the model.

***Experiment 6: Retraining 3D UNet of UNet+WS pipeline by augmenting with both over and under-exposure***

In this experiment, the training dataset for the 3D UNet model was augmented with both images with partial over and under-exposure effects. Also for testing, the same test set was used as in all the experiments above. The results from this experiment are also shown in S3 Fig above. The plot shows that this augmentation improves the accuracy for the over-exposed images whereas those for normal and underexposed ones remains the same as the original results (i.e from model trained on normal images only).

Thus the augmentation with under-exposed images (Experiment 5) is the most effective one for this model as revealed from the experiments. This augmentation produces an overall improvement in the segmentation quality of the UNet+WS pipeline.

***Observations***

The main observation from these experiments is that data augmentations could indeed help in improving the performance of deep learning based segmentation pipelines.The Plantseg pipeline results could be improved overall by adding both over and underexposure artifacts in the training set and the UNet+WS results could be improved using underexposure artifact as training data augmentation. However, it is also seen that different deep learning pipelines behave differently in response to data augmentations. While some pipelines may show improved results upon adding certain artifacts for augmenting the training dataset, another pipeline could be adversely affected by the same augmentation. Thus the best training dataset for each pipeline needs to be determined experimentally by observing their response to different training data augmentation strategies.
