# Supplementary figures and images for: Benchmarking of deep learning algorithms for 3D instance segmentation of confocal image datasets
Source: PLoS Comput Biol. 2022 Apr 14;18(4):e1009879. doi: 10.1371/journal.pcbi.1009879 (PMC9009699; doi:10.1371/journal.pcbi.1009879)

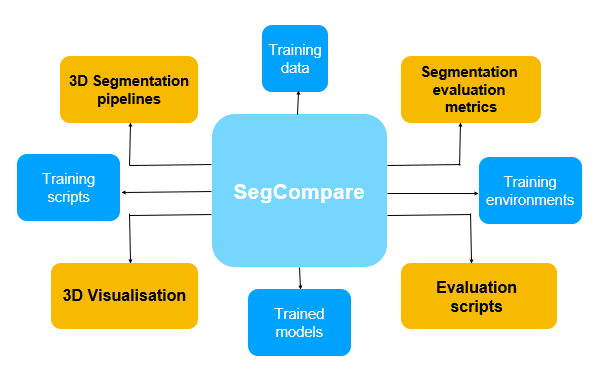

Supplement: S1 Fig — (TIFF) [file pcbi.1009879.s006.tiff]

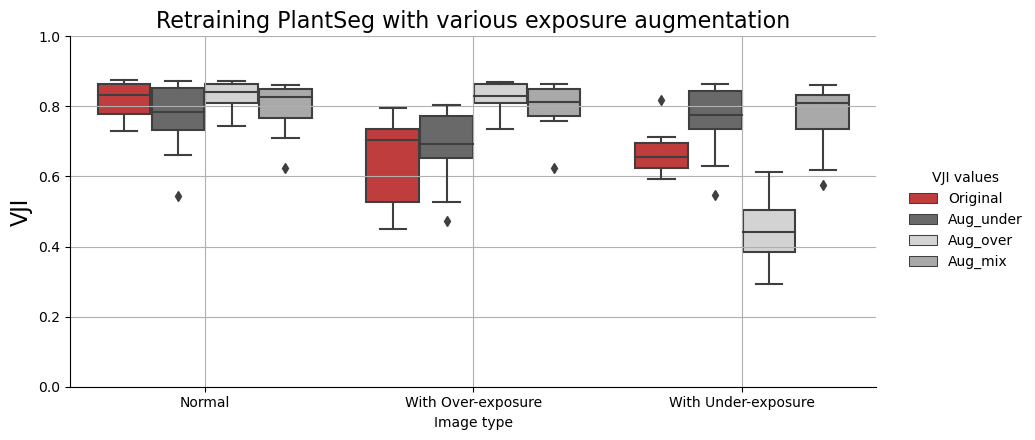

Supplement: S2 Fig — (TIF) [file pcbi.1009879.s007.tif]

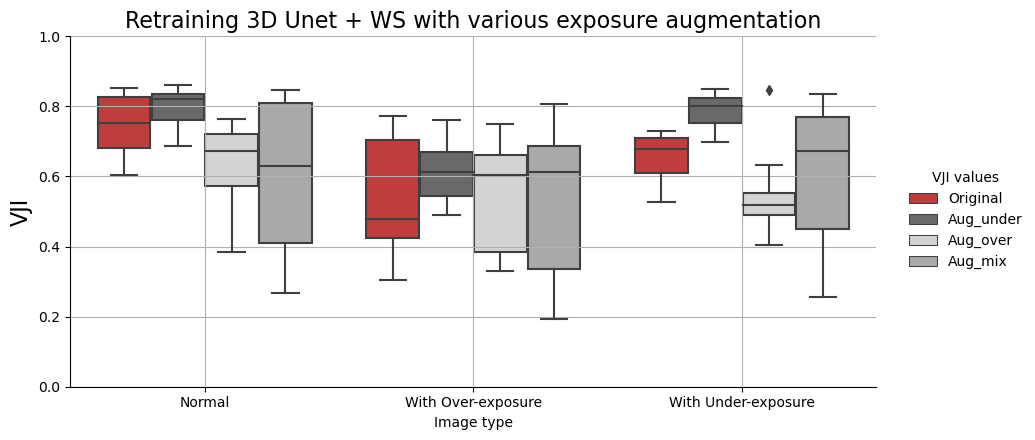

Supplement: S3 Fig — (TIF) [file pcbi.1009879.s008.tif]
